# ERK signaling controls productive HIF‐1 binding to chromatin and cancer cell adaptation to hypoxia through HIF‐1α interaction with NPM1

**DOI:** 10.1002/1878-0261.13080

**Published:** 2021-09-09

**Authors:** Kreon Koukoulas, Antonis Giakountis, Angeliki Karagiota, Martina Samiotaki, George Panayotou, George Simos, Ilias Mylonis

**Affiliations:** ^1^ Laboratory of Biochemistry Faculty of Medicine University of Thessaly Biopolis Greece; ^2^ Department of Biochemistry and Biotechnology University of Thessaly Biopolis Greece; ^3^ Institute for Bio‐innovation BSRC ‘Alexander Fleming’ Vari Greece; ^4^ Gerald Bronfman Department of Oncology Faculty of Medicine McGill University Montreal Canada

**Keywords:** cancer, HIF, hypoxia, NPM1, nucleophosmin

## Abstract

The hypoxia‐inducible factor HIF‐1 is essential for oxygen homeostasis. Despite its well‐understood oxygen‐dependent expression, regulation of its transcriptional activity remains unclear. We show that phosphorylation by extracellular signal‐regulated kinases1/2 (ERK1/2), in addition to promoting HIF‐1α nuclear accumulation, also enhances its interaction with chromatin and stimulates direct binding to nucleophosmin (NPM1), a histone chaperone and chromatin remodeler. NPM1 is required for phosphorylation‐dependent recruitment of HIF‐1 to hypoxia response elements, its interaction with acetylated histones, and high expression of HIF‐1 target genes under hypoxia. Transcriptome analysis revealed a significant number of hypoxia‐related genes commonly regulated by NPM1 and HIF‐1. These NPM1/HIF‐1α co‐upregulated genes are enriched in three different cancer types, and their expression correlates with hypoxic tumor status and worse patient prognosis. In concert, silencing of NPM1 expression or disruption of its association with HIF‐1α inhibits metabolic adaptation of cancer cells and triggers apoptotic death upon hypoxia. We suggest that ERK‐mediated phosphorylation of HIF‐1α regulates its physical interaction with NPM1, which is essential for the productive association of HIF‐1 with hypoxia target genes and their optimal transcriptional activation, required for survival under low oxygen or tumor growth.

## Graphical abstract

NPM1 marks and may organize hypoxia‐inducible promoters. Upper panel: In the absence of ERK‐mediated phosphorylation HIF‐1α binding to HRE is weak resulting in basal levels of transcriptional activity. Bottom panel: In cells with elevated ERK1/2 activity phosphorylated HIF‐1α can then bind to NPM1 and mediate stable association of HIF‐1 with a neighboring HRE resulting in maximal activation of transcription.

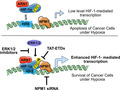

**Abbreviations**
ARNT: aryl hydrocarbon receptor nuclear translocator
DLBC: lymphoid neoplasm diffuse large B‐cell lymphoma
ERK1/2: extracellular signal‐regulated kinases1/2
ETD: ERK targeted domain
FRAP: fluorescence recovery after photobleaching
GBM: glioblastoma multiforme
HIF: hypoxia‐inducible factor
HRE: hypoxia response elements
LLPS: liquid–liquid phase separation
NES: nuclear export signal
NPM1: nucleophosmin
THYM: thymoma

## 1 Introduction

Cells exposed to hypoxia undergo a series of changes that allow for their metabolic adaptation and survival. In cancer cells, these changes facilitate tumor progression, metastasis, and resistance to drugs [1]. Adaptation to hypoxia at the transcriptional level is accomplished through activation of the heterodimeric Hypoxia‐inducible factors (HIFs) [2]. They consist of a stably expressed HIF‐β subunit or aryl hydrocarbon receptor nuclear translocator (ARNT) and an oxygen‐regulated HIF‐α subunit. In oxygenated cells, HIF‐α is modified by oxygen‐sensing prolyl‐hydroxylases (PHDs), which leads to association with the von Hippel–Lindau protein (pVHL) and proteasomal degradation [3]. In addition, asparagine hydroxylase factor inhibiting HIF‐1α (FIH) regulates HIF activity by inhibiting HIF‐α in association with CBP/p300 [4]. Under hypoxia, hydroxylation is impaired, HIF‐α escapes degradation and translocates into the nucleus where it binds to ARNT and hypoxia response elements (HREs), thereby inducing expression of target genes. HIF‐α is often overexpressed in human cancers as a result of local hypoxic microenvironment or oncogenic transformation and is associated with poor prognosis. HIF‐1α is expressed in most cells while expression of HIF‐2α is tissue and cell‐type specific. HIF‐1 and HIF‐2 have distinct as well as common targets [5]. HIFs are also implicated in obesity, nonalcoholic fatty liver disease, pulmonary hypertension, atherosclerosis, and other pathologies [2]. However, despite the undeniable importance of HIFs and the wealth of data for their oxygen‐dependent activation, there is, relatively, little information on events taking place downstream of HIF‐α stabilization and, especially the interaction between HIFs and chromatin or the transcriptional apparatus.

Hypoxia‐inducible factor‐1α, in particular, is affected by oxygen‐independent post‐translational mechanisms, including phosphorylation or/and interaction with other proteins [6, 7]. We have previously reported that CK1δ phosphorylates HIF‐1α on Ser247 and inhibits its association with ARNT and HIF‐1 activity, whereas phosphorylation of HIF‐1α by extracellular signal‐regulated kinases 1/2 (ERK1/2) on Ser641/643 (inside a domain termed ERK Targeted Domain; ETD) stimulates HIF‐1 activity [8, 9]. ETD phosphorylation by ERK1/2 masks an atypical nuclear export signal (NES) and, thereby, promotes nuclear HIF‐1α accumulation and HIF‐1 activity, while absence of this phosphorylation leads to translocation of HIF‐1α onto the mitochondria, where it forms a complex with antiapoptotic function [9, 10]. Interestingly, HIF‐1α‐derived cell permeable TAT‐ETD‐FLAG peptides that harbor the phosphorylation sites or a phosphomimetic mutation, when introduced into Huh7, HeLa, or HepG2 cells, could impair endogenous HIF‐1 transcriptional activity and cell survival under hypoxia [11].

Driven by the observation that the phosphorylated form of HIF‐1α ETD limits its nuclear mobility, suggesting stronger association with chromatin, we sought to identify its nuclear interacting partners. This quest led to the identification of NPM1 and the demonstration of the significance of the ETD/NPM1 interaction for the cellular adaptation to low oxygen conditions and its regulation.

## 2 Methods

### 2.1 Plasmids and protein expression

Plasmids encoding GST‐tagged NPM1 forms B23.1, B23.2, ΔC, ΔN, and CR were a generous gift from M. Okuwaki (Faculty of Medicine, University of Tsukuba, Japan) [12, 13]. All other plasmids have been previously described [8, 9, 11]. Recombinant proteins GST‐HIF‐1α(348–826) GST‐WT, GST‐SA, GST‐SE and GST‐ETD GST‐WT, GST‐SA, GST‐SE were expressed and purified as previously described [9]. To bacterially express the different GST‐NPM1 forms, plasmids encoding for GST‐NPM1 B23.1, GST‐B23.2, GST‐ΔC, GST‐ΔN, and GST‐CR were transformed into the BL21 (RIL) strain of *Escherichia coli*. Transformed bacteria were grown in LB Broth at 37 °C until OD600 reached 0.5 followed by addition of 0.1 mm isopropyl β‐d‐1‐thiogalactopyranoside (IPTG) at 22 °C for 2 h to induce protein expression. To purify GST‐tagged proteins, bacteria were lysed by sonication (Vibra‐Cell, Sonics and Materials, Newtown, CT, USA) in a buffer containing 20 mm Tris/HCl (pH 7.6), 0.1% Triton X‐100, 150 mm NaCl, 5 mm MgCl_2_, 1 mm DTT, and 0.1 mm AEBSF. The lysates were incubated with Glutathione (GSH)‐agarose (MACHEREY‐NAGEL, Dueren, Germany) under rotation for 1 h at 4 °C, and bound proteins were eluted by 10 mm reduced glutathione in 25 mm Tris‐HCl (pH 8.5). When required, GST‐tagged proteins were processed with His‐TEV as previously described [11] to remove the GST moiety. All protein constructs used in this study are schematically shown in Fig. S1.

### 2.2 Cell culture, transfections, and reporter gene assays

Human HeLa (CVCL_0058) or (CVCL_R965; acquired from ATCC, Manassas, VA, USA), and Huh7 (CVCL_0336) cells (a kind gift from M. Muckenthaler, University of Heidelberg; originally purchased from the Japanese Collection of Research Bioresources), regularly tested for mycoplasma, were cultured in DMEM (Biosera, Nuaille, France) containing heat‐inactivated 10% FBS and 100 U·mL^−1^ penicillin‐streptomycin (Biochrom, Berlin, Germany) in a CO_2_ humidified incubator at 37°C or (for hypoxia) in an INVIVO2 200 hypoxia workstation (Baker Ruskinn, Sanford, ME, USA) at 1% O_2_. Huh7 cells were used in fluorescence recovery after photobleaching (FRAP) assays due to their superior nuclear fluorescent signals and in ChIP experiments because of their previously well‐characterized *LPIN1* and *AGPAT2* promoters. HeLa cells were used in all other experiments due to their higher proliferation rates. Results were consistent for both cell lines. To inactivate the ERK1/2 pathway, cells were treated for 16 h with 5 or 10 μm U0126 (as indicated; MEK inhibitor; Cell Signaling, Danvers, MA, USA) or were serum‐deprived. Cells were transiently transfected with 10 μg plasmid DNA or 20 nm siRNAs using the JetPRIME^®^ Polyplus reagent (Polyplus, Strasbourg, France) or VIROMER^®^BLUE (BioNTech, Mainz, Germany). Details of siRNAs are shown in Table S1. Reporter gene assays were carried out as previously described [9].

### 2.3 *In vitro* binding assays and immunoprecipitation

*In vitro* binding assays using as baits GST‐tagged ETD, HIF‐1α (348–826), NPM1, and their mutant forms and as pray HeLa protein extracts or purified proteins, as well as IP of HIF‐1α, NPM1, and GFP‐ or Flag‐tagged proteins using the antibodies shown in Table S2, were performed as previously described [10].

### 2.4 Western blotting and immunofluorescence microscopy

Protein analysis by immunoblotting, detection by immunofluorescence microscopy, and visualization/quantification of results were carried out as previously described [9] using the antibodies presented at Table S2. Lipid droplet staining was performed using Nile Red (0.1 mg in PBS; Sigma‐Aldrich, St Louis, MO, USA) for 15 min before mounting on slides [14].

### 2.5 Live cell imaging and fluorescence recovery after photobleaching

Analysis of Huh7 cells expressing GFP or GFP‐HIF‐1α phosphorylation mutants by live cell imaging and FRAP were performed as previously described [8]. Quantitative analysis was performed using easyFRAP [15].

### 2.6 Trypsinization, LC‐MS/MS, and data analysis

In‐gel tryptic digestion of proteins, LC‐MS/MS, and data analysis was performed according to standard procedures [16] and as described in detail previously [10].

### 2.7 Chromatin immunoprecipitation

Chromatin immunoprecipitation experiments of Huh7 cells were performed as previously described [14] using antibodies shown in Table S2. In sequential ChIP (ChIP‐re‐ChIP) experiments, first chromatin immunoprecipitates (IP) were eluted with 1× TE buffer containing 2% SDS and 15 mm DTT, the eluates were diluted 10‐fold in IP buffer, and they were then processed for the second IP step as for the first. Amplification of the −2916 to −2686 region of the *hLPIN1* promoter or the different HRE regions of the AGPAT2 promoter (Table S3), subsequent analysis, and quantification was performed as previously described [14, 17].

Amplification of the promoter regions of *HSPB1, CASP9, and HAMP* (Table S3), analysis, and quantification were performed as previously described [18, 19, 20].

### 2.8 RNA extraction and quantitative RT‐PCR

Total RNA isolation was performed by using the NucleoZOL reagent (MACHEREY‐NAGEL, Germany), and cDNAs were synthesized by M‐MuLV Reverse Transcriptase (New England BioLabs, Ipswich, MA, USA) or the High‐Capacity Reverse Transcription Kit (Applied Biosystems, Foster City, CA, USA). Quantitative PCR was carried out in a LightCycler® 96 System (Roche, Basel, Switzerland), using the KAPA SYBR FAST qPCR kit (Kapa Biosystems, Wilmington, MA, USA). Primers for amplification of cDNAs are shown in Table S3.

### 2.9 Quant‐seq analysis

For Quant‐seq, poly‐A isolated RNA was sequenced with an Ion Proton™ System. Read mapping was performed tophat2 with default settings. Unmapped reads were re‐mapped with Bowtie2 against the hg19 genome with the very‐sensitive flag and merged with the initial mappings. Statistical analysis was performed with DESeq through the Bioconductor package metaseqR [21]. Differentially expressed genes presented a binomial test *P*‐value < 0.05 and fold change (for each contrast) > 0.58 or < −0.58 in log_2_ scale. Volcano plots and heatmaps were performed in r (The R Foundation, Indianapolis, IN, USA). Venn diagrams were performed with Venny, gene ontology with genecodis [22] and statistical analysis of gene overlaps with hypergeometric tests in r.

### 2.10 Cell death, annexin V, and TUNEL assays

Cell death was determined by the LDH cytotoxicity detection kit (Takara‐Clontech, Mountain View, CA, USA). Phosphatidylserine translocation and DNA fragmentation were detected by using the ‘CF555 Annexin V and PI Apoptosis Assay’ Kit (Biotium, Fremont, CA, USA) and ‘In situ Apoptosis Detection’ Kit (Takara‐Clontech, Mountain View, CA, USA), respectively. Images were taken on a Zeiss Axio Imager.Z2 microscope equipped with AxioCam MRm sensor and 20× objective.

### 2.11 Datasets, gene expression, and survival analysis

Transcriptome data and clinicopathological information were analyzed by the Gene Expression Profiling Interactive Analysis 2 (GEPIA2) online platform [23] utilizing The Cancer Genome Atlas (TCGA) and Genotype‐Tissue Expression (GTEx) datasets: http://gepia2.cancer‐pku.cn/#dataset. Analysis included gene expression profiling of tumor and paired normal tissues, gene boxplot expression analysis (*P* < 0.05 deemed significant), single or multigene Kaplan–Meier curves and survival maps using Mantel–Cox test, and correlation analysis between genes or gene signatures by using Spearman test, all performed as described by [23]. To create a hypoxia gene signature, the Gene Set Enrichment Analysis library containing 200 hypoxia‐upregulated genes (https://www.gsea‐msigdb.org/gsea/msigdb/cards/HALLMARK_HYPOXIA), was used. Following, we performed network analysis using STRING (https://string‐db.org/cgi/input.pl) in order to identify genes with the strongest functional correlation with HIF‐1 pathway [24].

### 2.12 Image analysis and statistical analysis

Fluorescence and colocalization quantification was performed using plugins of the imagej public domain software (v.1.51g, NIH, Bethesda, MD, USA) as previously described [10].

Statistical variance between two groups of values was calculated using the prism software (GraphPad; version 5.04) and applying Student's *t*‐test (two‐tailed) or by one‐way analysis of variance (ANOVA) within multiple groups; *P* < 0.05 was deemed statistically significant (as indicated).

## 3 Results

### 3.1 HIF‐1α phosphorylation by ERK1/2 stimulates HIF‐1α binding to chromatin components

#### 3.1.1 ERK‐mediated phosphorylation decreases the intranuclear mobility of HIF‐1α

**Fig. 1:**
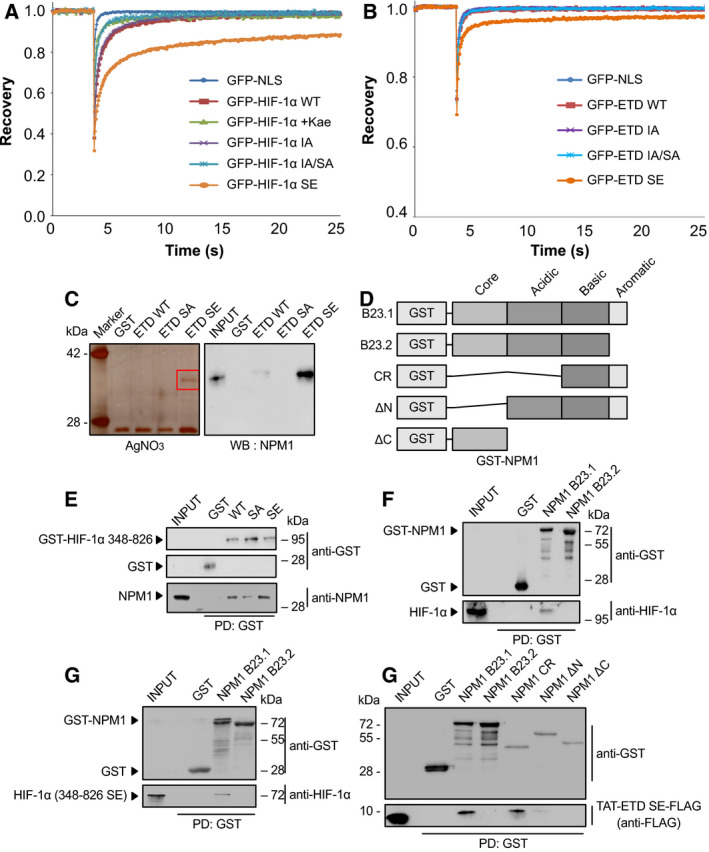
Identification of NPM1 as a phosphorylation‐dependent and direct interaction partner of HIF‐1α. (A,B) Phosphorylation of HIF‐1α by ERK increases its affinity for intranuclear immobile elements. Huh7 cells overexpressing the indicated full‐length GFP‐tagged (A) HIF‐1α or (B) HIF‐1α ETD forms were grown under normoxia and analyzed with FRAP 24 h post‐transfection. When needed, cells were treated with 50 μμ of kaempferol for 4 h as specified (WT+kae), in order to inhibit ERK activation. Curves represent the mean corrected fluorescence intensities over time. Curves represent the mean of two independent experiments (number of analyzed cells (*n*) are given in Table S4; ± SD for each curve is shown in Figs S2 and S3. (C) Analysis of HeLa cell proteins bound to different recombinant, purified, and immobilized on GSH‐agarose mutant forms of the GST‐HIF‐1α(ETD) (as indicated) after their elution by TEV‐mediated cleavage of the GST moiety. Left panel: Analysis by SDS/PAGE/AgNO_3_ staining (red square specifies gel section analyzed by mass spectrometry). Right panel: Analysis by western blotting (WB) with an anti‐NPM1 antibody. Images are representative of three independent experiments (see also Fig. S4A). (D) Schematic representation of domain structure of full‐length GST‐tagged NPM1 isoforms (B23.1 or B23.2) or their truncation forms CR, ΔN, and ΔC used in the following *in vitro* binding assays. (E) Soluble HeLa protein extracts (INPUT) were mixed with GSH‐agarose beads carrying GST or different mutant forms of GST–HIF‐1α(348–826) (as indicated), and bound proteins (PD: Pull‐Down) were analyzed by immunoblotting using antibodies against GST or NPM1 proteins. (F–H) GSH‐agarose beads carrying GST alone or different bacterially expressed and purified forms/domains of GST‐NPM1 were mixed with soluble protein extracts from hypoxic HeLa cells (F; INPUT) or bacterially expressed and purified phosphomimetic mutant HIF‐1α(348–826)SE (G; INPUT) or bacterially expressed and purified phosphomimetic TAT‐ETD‐SE‐FLAG peptide (H; INPUT) and bound proteins (PD: Pull‐Down) were analyzed by immunoblotting using antibodies against GST, HIF‐1α, and Flag (as indicated). Panels in C, E, F, G, H show single blot areas that correspond to the indicated molecular weight marker and were cut after blotting for analysis with different antibodies; images in E, F, G, H are representative of two independent experiments.

To test whether phosphorylation by ERKs may influence retention of HIF‐1α inside the nucleus, FRAP experiments were performed in Huh7 cells transiently expressing wild‐type (WT) GFP‐HIF‐1α or mutant forms in the absence or presence of kaempferol that inactivates ERK [25]. The mutant forms, shown schematically in Fig. S1, included SE, carrying a phosphomimetic mutation (Ser641 to Glu; previously shown to be nuclear); SA with mutations that abolish phosphorylation (Ser641/643 to Ala; previously shown to reside predominantly outside the nucleus); IA with mutations that destroy the NES (Ile637/639 to Ala; previously shown to persistently reside inside the nucleus even in the absence of ERK phosphorylation); and IA/SA with mutations that destroy both the NES and the ERK phosphoacceptor sites (Ile637/639 to Ala and Ser641/643 to Ala; previously shown to be nuclear, although its lacks phosphorylation). No FRAP results could be obtained for the phospho‐deficient SA form as the nuclear fluorescence signal was too low (Fig. S2). The resulting FRAP recovery curves (Fig. 1A) and quantitative analysis using easyFRAP [15] for the other GFP‐HIF‐1α forms and GFP‐NLS (used as a freely diffusible nuclear control protein; Table S4) gave the following results. As expected, GFP‐NLS exhibited fast and full recovery of fluorescence, the highest diffusion coefficient (*D*_eff_), and mobile fraction (*f*_mob_) and the lowest half‐maximal recovery time (*t*_1/2_). WT GFP‐HIF‐1α and the mutant IA form, both able to be reversibly phosphorylated by ERKs, showed similar recovery curves and *D*_eff_, *f*_mob_, and *t*_1/2_ values (with insignificant differences, *P* > 0.05), suggesting that disruption of the NES restricts HIF‐1α inside the nucleus but does not affect its intranuclear mobility. In contrast to the WT and IA forms, both kaempferol treatment of cells expressing the WT form (WT + Kae) and mutation of the ERK sites in the IA/SA form resulted in faster fluorescence recovery, significantly higher D_eff_ and lower t_1/2_ compared to WT (*P* < 0.05 for WT+Kae and *P* < 0.001 for IA/SA), suggesting that inhibition of phosphorylation reduces the affinity of HIF‐1α for immobile nuclear elements. This was reinforced by the phosphomimetic SE form, which exhibited the exact reverse: slower and decreased fluorescence recovery, remarkably lower *D*_eff_ and *f*_mob_, and much higher *t*_1/2_ compared to the WT form (*P* < 0.001), suggesting that irreversible phosphorylation of the ERK site renders a significant fraction of HIF‐1α virtually immobile inside the nucleus by strong tethering to chromatin or nuclear matrix.

#### 3.1.2 A phosphomimetic mutation decreases the intranuclear mobility of HIF‐1α ETD

The FRAP results with the full‐length HIF‐1α SE form were largely confirmed by using Huh7 cells expressing the 43‐amino acid long ETD (amino acids 616–658) as an independent GFP‐fusion peptide in its WT or mutant forms (Fig. 1B, Fig. S3). Specifically, the phosphomimetic mutation in ETD (ETD‐SE) caused lower mobility compared to the other ETD forms (*P* < 0.05), which exhibited similar diffusion kinetics (Table S4). These results suggested that ETD mediates phosphorylation‐dependent tethering of HIF‐1α onto nuclear/chromatin structures. As the ETD lies away and is distinct from the DNA binding, heterodimerization, and transactivation domains, its tethering properties could only be explained by specific and strong interaction of its phosphorylated form with certain, as yet unknown, nuclear components.

### 3.2 HIF‐1α carrying a phosphomimetic mutation binds directly to the carboxy‐terminal domain of NPM1

#### 3.2.1 Identification of NPM1 as an interacting protein of HIF‐1α ETD

In order to identify phosphorylation‐dependent HIF‐1α ETD interactions that could explain its affinity for chromatin/nuclear structures, different forms of GST‐tagged ETD peptides that had been expressed in *E. coli*, purified, and immobilized on GSH‐agarose beads were used as baits in pull‐down assays with total HeLa cell protein extracts. After elution of bound proteins by GSH, a protein with an apparent molecular mass 36 kD could be detected to bind specifically to the phosphomimetic GST‐ETD‐SE form but not to the phospho‐deficient GST‐ETD‐SA form or the WT GST‐ETD form, which as being recombinant should be in the nonphosphorylated form (Fig. S4A). To facilitate identification of the 36 kDa protein by mass spectroscopy, the experiment was repeated with elution of the bound proteins by TEV‐mediated cleavage of the GST moiety of the immobilized baits, which revealed again the presence of the 36 kDa protein in specific association with ETD‐SE (Fig. 1C; left). Subsequent mass spectrometry analysis of the 36 kDa band, identified it as nucleophosmin (NPM1, B23; Table S5). Its identity was further confirmed by immunoblotting analysis using an anti‐NPM1 antibody (Fig. 1C; right), which also revealed weak binding of NPM1 to WT ETD.

Nucleophosmin is a widely expressed, mostly nucleolar, protein involved in diverse nuclear functions [26, 27]. Interestingly, NPM1 expression can be stimulated under hypoxia by HIF‐1 [28], and like HIF‐1α, NPM1 is often overexpressed in solid tumors [29]. NPM1 consists of a N‐terminal oligomerization (core) domain, an acidic domain harboring ribonuclease and histone chaperone activity and a basic C‐terminal domain containing an aromatic stretch responsible for nucleic acid binding, especially G‐rich DNA, nucleolar localization, and ATP binding (Fig. 1D). NPM1 isoform B23.1 is the most abundant form, while B23.2 is a common splicing variant that lacks the C‐terminal aromatic stretch [30].

#### 3.2.2 The C‐terminal domain of NPM1 is required for direct binding to HIF‐1α ETD *in vitro*

To verify the interaction between HIF‐1α ETD and NPM1, pull‐down assays with HeLa cell extracts were repeated using as baits a larger recombinant *E. coli* expressed and purified GST‐tagged part of HIF‐1α comprising amino acids 348–826, in WT or mutant forms (Figs S1 and S4B). The results shown in Fig. 1E confirmed the stronger binding of GST‐HIF‐1α(348–826) to native HeLa NPM1 in the presence of the phosphomimetic mutation (SE) as compared to the WT or SA mutant. To further test whether the identified interaction of the ETD region of HIF‐1α with NPM1 was direct and not mediated by another, as yet unknown, protein, recombinant *E. coli* expressed and purified GST‐tagged NPM1 splice variants B23.1 and B23.2 [12, 13] (Fig. S4C) were immobilized on GSH‐agarose beads and used as baits in pull‐down assays with either hypoxic HeLa cell extracts (expressing endogenous native HIF‐1α) or recombinant *E. coli* expressed and purified HIF‐1α(348–826)SE (Fig. S4B). As shown in Fig. 1F,G, both native HeLa HIF‐1α and purified recombinant HIF‐1α(348–826)SE bound only to the B23.1 and not to the B23.2 form of recombinant NPM1, suggesting that the C‐terminal aromatic domain of NPM1 is necessary for the formation of the NPM1/HIF‐1α complex. To confirm this, additional recombinant *E. coli* expressed and purified GST‐tagged NPM1 truncation mutants (Fig. 1D, Fig. S4C; [12]) were tested for binding to the TAT‐ETD(SE)‐FLAG peptide [11], which, due to its small size (Fig. S4B), could be expressed in *E. coli* and purified in a more stable and abundant form than HIF‐1α(348–826)SE. As shown in Fig. 1H, TAT‐ETD(SE)‐FLAG is readily associated with the GST‐B23.1 NPM1 variant and GST‐NPM1(CR) but not with the GST‐B23.2 variant or GST‐NPM1(ΔC). Only weak binding of TAT‐ETD(SE)‐FLAG to GST‐NPM1(ΔN) was detected, probably because of conformational issues with the construct.

Taken together, the results of the *in vitro* binding assays showing specific association between recombinant *E. coli* expressed and purified protein constructs of HIF‐1α and NPM1 as well as between recombinant HIF‐1α or NPM1 constructs and endogenous native HeLa NPM1 or HIF‐1α, respectively, are compatible with direct binding of HIF‐1α to NPM1 mediated by the phosphorylated form of ETD and the C‐terminal aromatic domain of NPM1.

### 3.3 NPM1 association with HIF‐1α is regulated by ERK1/2 and facilitates HIF‐1 transcriptional activity

#### 3.3.1 Phosphorylation‐dependent physical association between HIF‐1α and NPM1 in cells under hypoxia

**Fig. 2:**
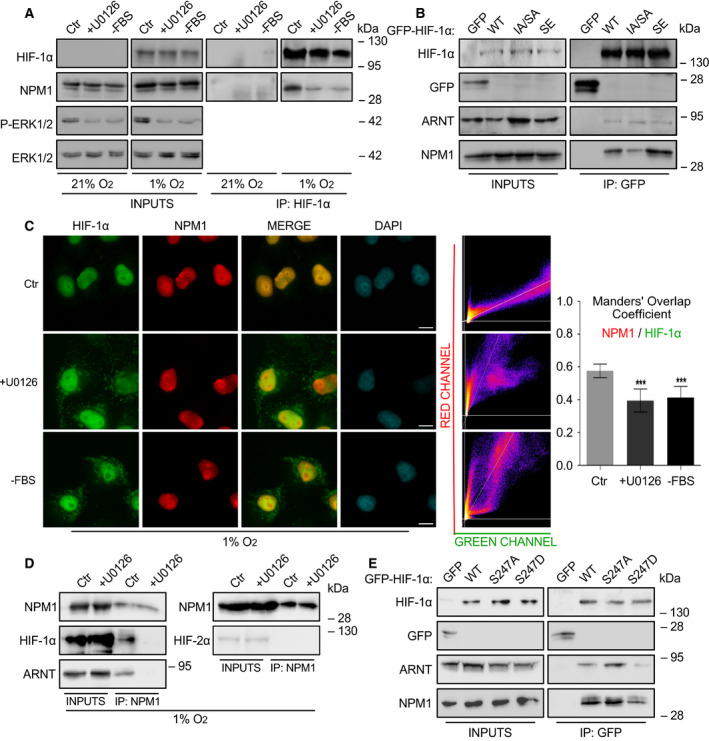
The association of HIF‐1α with NPM1 inside HeLa cells is regulated by ERK1/2. (A) Soluble proteins (INPUTS) or anti‐HIF‐1α IP of HeLa cells grown at 21% or 1% O_2_ for 16 h, untreated (Ctr) or treated with 5 μm U0126 (+U0126) or deprived of serum (‐FBS) were analyzed by immunoblotting using antibodies against HIF‐1α, NPM1, phospho‐ERK1/2 and ERK1/2 as indicated. (B) Soluble extracts (INPUTS) or anti‐GFP IP of HeLa cells transiently expressing GFP or GFP‐tagged full‐length HIF‐1α WT, IA/SA, SE forms were analyzed at 20 h post‐transfection by immunoblotting using antibodies against HIF‐1α, GFP, ARNT, and NPM1 as indicated. (C) Immunofluorescence microscopy analysis of cells grown at 1% O_2_ and treated as in (A) using antibodies against HIF‐1α (Green) or NPM1 (Red). Nuclei were stained with DAPI (Cyan; Scale bars: 10 μm). Middle panels are scatterplots of pixel intensities of HIF‐1α and NPM1 signals. Graph shows the Manders' overlap coefficient as measured in nuclei‐restricted fluorescence in 35 cells from two independent experiments in each condition ± SEM (***P < 0.001; Statistical variance between two groups of values was calculated by two‐tailed Student's *t*‐test). (D) Soluble proteins (INPUTS) or anti‐NPM1 IP of HeLa cells incubated at 1% O_2_ for 16 h and treated (+U0126) or not (Ctr) with 5 μm U0126 were analyzed by SDS/PAGE and western blotting using antibodies against NPM1, HIF‐1α, and ARNT as indicated. (E) Soluble extracts (INPUTS) or anti‐GFP IP of HeLa cells transiently expressing GFP or GFP‐tagged full‐length HIF‐1α WT, S247A, S247D forms were analyzed at 20 h post‐transfection by immunoblotting using antibodies against HIF‐1α, GFP, ARNT, and NPM1 as indicated. Certain panels in A, B, D, E show single blot areas that correspond to the indicated molecular weight marker and were cut after blotting for analysis with different antibodies; images in A, B, D, E are representative of three (A) or two (B, D, E) independent experiments.

It was then tested whether the interaction between HIF‐1α and NPM1 also occurs inside living cells under the control of the ERK1/2 pathway using immunoprecipitation (IP) experiments. Endogenous NPM1 was associated with HIF‐1α in HeLa cells grown under hypoxic conditions (Fig. 2A). Importantly, their association was reduced after ERK1/2 pathway inactivation by either using the U0126 MEK‐selective inhibitor or withdrawing the serum from the growth medium of the cells. To corroborate that the requirement of ERK1/2 activation for the HIF‐1α/NPM1 association reflects the need for modification of the HIF‐1α ERK sites, mutant forms of GFP‐HIF‐1α were immunoprecipitated from overexpressing HeLa cells using an anti‐GFP antibody. NPM1 was readily detectable within IPs of WT HIF‐1α, while its association with the phospho‐deficient HIF‐1α IA/SA mutant was much weaker (Fig. 2B). In contrast, association of NPM1 with the phosphomimetic HIF‐1α SE mutant was significantly stronger.

#### 3.3.2 Phosphorylation‐dependent colocalization of HIF‐1α and NPM1 in nuclei of cells under hypoxia

To substantiate the above, both endogenous HIF‐1α and NPM1 were localized in hypoxic HeLa cells by immunofluorescence microscopy. As shown in Fig. 2C, there is substantial colocalization of the two proteins inside the cell nucleus and especially at the nucleolar periphery. This colocalization is specific as it was largely abolished upon inhibition of the ERK1/2 pathway, concomitant with the translocation of a significant HIF‐1α fraction outside the nucleus. It is worth mentioning that NPM1 localization was not affected by either hypoxic treatment or inhibition of ERK1/2 activation and remained nuclear with enrichment at the nucleolar periphery under all conditions (Compare Fig. 2C with Fig. S5).

Taken together, the IP and microscopic data from HeLa cells are in full agreement with the *in vitro* binding data and show that HIF‐1α association with NPM1 at specific intranuclear sites is under the control of ETD phosphorylation by ERK1/2.

#### 3.3.3 NPM1 associates with the HIF‐1α/ARNT heterodimer and increases its transcriptional activity

The question addressed next was if and how association with NPM1 affects the function of HIF‐1α. In order for HIF‐1α to exert its transactivation activity, it must form a DNA‐binding heterodimer with ARNT. Initially, and in accordance with HIF‐1α immunoprecipitations, HIF‐1α could also be coimmunoprecipitated with NPM1 from hypoxic HeLa cells but not in the presence of the MEK inhibitor (Fig. 2D, left). This experiment also established that NPM1 does not detectably associate with the second HIF‐α isoform, HIF‐2α, as it could be expected by the fact that the amino acid sequence of HIF‐1α ETD is not conserved in HIF‐2α (Fig. 2D, right). Furthermore, the presence of ARNT in the NPM1 IP implied that HIF‐1α might preferentially bind to NPM1 after its association with ARNT (Fig. 2D, left). To further investigate how the formation of HIF‐1 heterodimer may influence the HIF‐1α/NPM1 association, Hela cells were transfected with GFP‐tagged HIF‐1α forms carrying mutations that abolish or mimic phosphorylation by CK1δ (Fig. S1), that either strengthen or weaken HIF‐1α interaction with ARNT, respectively [8]. Subsequent immunoprecipitation with an anti‐GFP antibody (Fig. 2E) revealed that the GFP‐HIF‐1α S247A mutant form that interacts stronger with ARNT also exhibits higher affinity for NPM1 compared to the GFP‐HIF‐1α S247D mutant that largely loses its association with both ARNT and NPM1. These results suggest that HIF‐1α binds to NPM1 while also in a complex with ARNT and makes likely that the association between NPM1 and HIF‐1 plays a role in HIF‐1‐mediated transcription of hypoxia target genes.

**Fig. 3:**
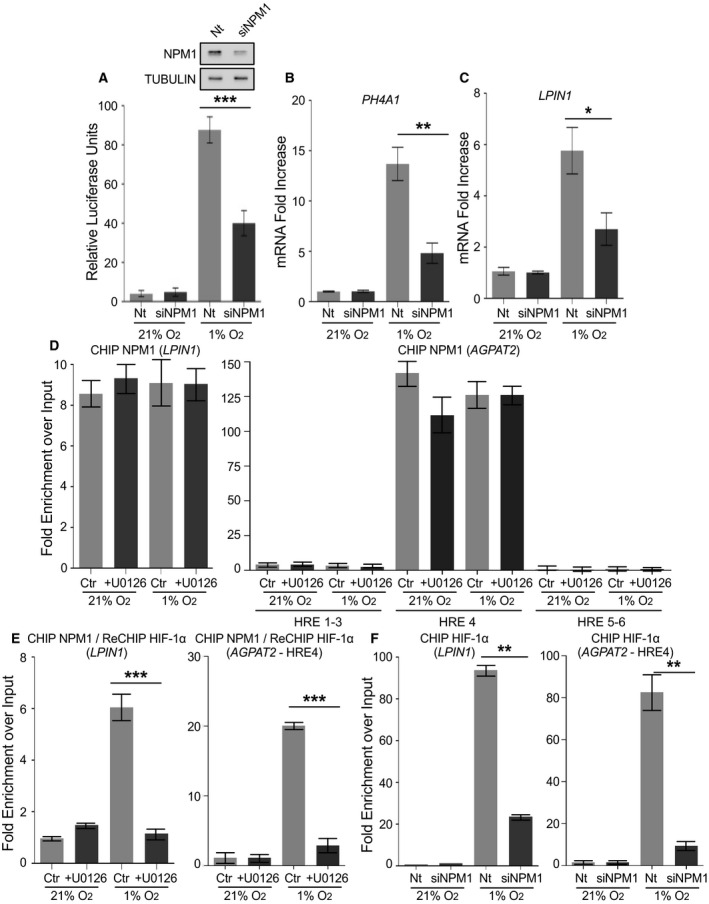
NPM1 is essential for HIF‐1 transcriptional activity and stable binding to HRE. (A) Transcriptional activity of HIF‐1 in HeLa cells, cotransfected with control (Nt) or NPM1 siRNA (siNPM1) and the pGL3–5HRE‐VEGF and pCI‐Renilla reporter plasmids and grown at 21% or 1% O_2_ for 16 h. Values were determined as ratio of firefly luciferase activity over renilla activity and represent the mean of two independent experiments performed in quadruplets ± SEM (***P < 0.001). Immunoblots shown as inset demonstrate NPM1 levels without (Nt) or after NPM1 silencing (siNPM1). Tubulin was used as loading control. (B, C) mRNA levels of HIF‐1 target genes *P4HA1‐1* (B) or *LPIN1* (C) as determined by RT‐PCR in HeLa cells treated with control (Nt) or NPM1 siRNA (siNPM1) and grown at 21% or 1% O_2_ for 16 h. Results are shown as fold increase in relation to respective normoxic conditions and represent the mean of two independent experiments performed in triplicates ± SD (*n* = 6; *P < 0.05, **P < 0.01). (D, E) RT‐PCR analysis of sequential chromatin immunoprecipitation (ChIP‐re‐ChIP) using first anti‐NPM1 (ChIP; D) and then anti‐HIF‐1α antibodies (re‐ChIP; E) from Huh7 cells grown at 21% or 1% O_2_ for 24 h with or without 10 μm U0126 (as indicated). Left panels in (D) and (E) show analysis using primers for the area of the *LPIN1* promoter containing a known HIF‐1 binding site (for details see also Fig. S6B). Right panels in (D) and (E) show analysis using primers for the areas of the *AGPAT2* promoter containing a known HIF‐1 binding site (HRE4) or nonfunctional HRE‐like sequences (HRE1–3, HRE5–6; for details see also Fig. S6B). (F) RT‐PCR analysis of ChIP with anti‐HIF‐1α antibodies from Huh7 cells treated with control (Nt) or NPM1 siRNA (siNPM1) for 24 h and incubated at 21% or 1% O_2_ for 8 h. Left panel: analysis using primers for the HIF‐1 binding site in the *LPIN1* promoter. Right panel: analysis using primers for the HIF‐1 binding site (HRE4) in the *AGPAT2* promoter. Results represent the mean of two independent experiments analyzed in triplicates ± SD (unpaired *t*‐test; *P < 0.05; **P < 0.01; ***P < 0.001).

Indeed, silencing of NPM1 expression in HeLa cells grown under hypoxia (Fig. 3A; insets) significantly lowered HIF‐1 transcriptional activity using a reporter gene assay (Fig. 3A; graph). Inhibition of HIF‐1 activity was also confirmed by showing that depletion of NPM1 in hypoxic HeLa cells also greatly reduced the expression of two specific HIF‐1 target genes, *PH4A1* ([31]; Fig. 3B) and *LPIN1* ([14]; Fig. 3C). In addition to *LPIN1* mRNA levels, Lipin1 protein levels were also reduced upon NPM1 silencing under hypoxia (Fig. S6A). Therefore, the phosphorylation‐dependent association between HIF‐1α ETD and NPM1 has important functional significance since NPM1 is essential for optimal transcriptional activity of HIF‐1 in HeLa cells.

### 3.4 NPM1 associates with HRE‐containing chromatin and enables stable binding of phosphorylated HIF‐1

#### 3.4.1 NPM1 specifically occupies HIF‐1‐binding sites on chromatin

In order to gain insight into the mechanism through which NPM1 stimulates HIF‐1 transcriptional activity, we analyzed two gene promoters (*LPIN1* and *AGPAT2)* that we previously characterized as specific HIF‐1 targets in Huh7 cells [14, 17]. *LPIN1* and *AGPAT2* promoters contain 8 and 6 predicted HRE‐like sequences, respectively, but only one of them was shown to be functional both in terms of driving HIF‐1‐dependent transcription and associating physically with HIF‐1α (shown schematically in Fig. S6B). We performed chromatin immunoprecipitation (ChIP) with anti‐NPM1 antibodies followed by re‐ChIP of the NPM1‐associated chromatin with anti‐HIF‐1α antibodies (i.e., ChIP‐re‐ChIP) in Huh7 cells grown either at normoxia or hypoxia and treated with or without U0126. The results of the first ChIP (anti‐NPM1) revealed that the functional HREs of the *LPIN1* and *AGPAT2* promoters that constitute HIF‐1 binding sites are enriched in the NPM1 ChIPs, irrespective of oxygen levels and ERK activation status, while DNA stretches containing the nonfunctional *AGPAT2* HRE‐like sequences (HRE1–3 and 5–6) were not found associated with NPM1 (Fig. 3D, Fig. S6C), suggesting that NPM1 is constitutively linked to two hypoxia‐inducible promoters and only to their functional ‘true’ HREs that serve as HIF‐1 binding sites. Analysis of the re‐ChIP (anti‐HIF‐1α) results showed that HIF‐1α co‐occupies with NPM1 the functional *LPIN1* or *AGPAT2* HREs only under hypoxia and only in the presence of active ERK1/2 (Fig. 3E, Fig. S6D), suggesting that phosphorylation of HIF‐1α by ERK1/2 enables binding to NPM1, which in turn promotes stable HIF‐1/HRE association and efficient gene activation.

To further verify the specificity of the interaction between NPM1 and chromatin containing HIF‐1 binding sites, we tested its association with four additional promoter areas (shown schematically in Fig. S7A) in Huh7 cells: the *HSPB1* gene promoter area containing two HRE sequences previously shown to serve as HIF‐1 binding sites (as positive control; [20]), an *HSPB1* gene promoter area lacking HRE‐like sequences (as negative control), the *HAMP* gene promoter area containing two HRE‐like sequences which, however, do not constitute true HIF‐1 binding sites (as negative control; [18]), and an area of the promoter of *CASP9*, which neither contains HRE‐like sequences nor is a known target of HIF‐1 (as negative control, [19]). The ChIP results from Huh7 cells with anti‐NPM1 antibodies showed association of NPM1 with the positive control promoter area (*HSPB1* HRE; Fig. S7B, top left panel) but no detectable binding to the three promoter areas used as negative controls (Fig. S7B, left panel HPSPneg; and S7C). Analysis of re‐ChIP with anti‐HIF‐1α antibodies confirmed that the HRE‐containing *HSPB1* gene promoter area, used as positive control for binding to NPM1, does indeed constitute a true HIF‐1 binding site under hypoxic and ERK1/2‐activating conditions (Fig. S7B, right panel), and is co‐occupied by both NPM1 and HIF‐1 just like the *LPIN1* and *AGPAT2* promoter areas analyzed above. Finally, the ChIP‐re‐ChIP experiment was repeated in the reverse order in Huh7 cells, that is, first ChIP with anti‐HIF‐1α antibodies followed by re‐ChIP of the HIF‐1α‐associated chromatin with anti‐NPM1 antibodies. The results showed that the HIF‐1‐binding sites of the *LPIN1* and *AGPAT2* promoters were also enriched in the NPM1 re‐ChIPs from the samples only containing HIF‐1‐associated chromatin (Fig. S7D). Therefore, all our ChIP results are consistent with specific association of NPM1 with active hypoxia‐targeted promoters and simultaneous occupation of HIF‐1 binding chromatin sites by both NPM1 and phosphorylated HIF‐1.

#### 3.4.2 NPM1 is required for stable binding of HIF‐1 onto HRE‐containing chromatin

Taking the results above (Fig. 3D,E) together with the requirement of NMP1 for HIF‐1 transcriptional activity (Fig. 3A–C), it can be suggested that phosphorylation of HIF‐1α by ERK1/2 enables its binding of NPM1, which in turn stabilizes HIF‐1 association with its target HRE and leads to efficient gene activation. This is indeed supported by ChIP experiments showing that binding of HIF‐1α to the *LPIN1* or *AGPAT2* functional true HREs was greatly inhibited when NPM1 expression was silenced in Huh7 cells (Fig. 3F, Fig. S6E). It should be noted here that this ChIP experiment was performed after 8 h incubation under hypoxia, a time point at which downregulation of NPM1 expression does not detectably affect cellular fitness, which starts decreasing by onset of apoptosis after at least 24 h of hypoxic treatment (see below; Fig. 5). Furthermore, NPM1 silencing in HeLa cells also resulted in reduced association of HIF‐1α with acetylated histone 4 (H4), a marker of active ‘open chromatin’ (Fig. S6F). Therefore, strong interaction of HIF‐1 with HRE‐containing active promoters requires the phosphorylation‐dependent association of HIF‐1α with the constitutively expressed and chromatin‐bound NPM1. In fact, as NPM1 expression has been previously shown to be induced by HIF‐1 and hypoxia [28], a finding in agreement with our data (see Fig. S6A,F), our results also suggest the operation of an ERK‐controlled positive feed‐forward mechanism, based on amplification of HIF‐1 activity following upregulation of NPM1 expression by HIF‐1 itself.

### 3.5 NPM1 is required for the cellular transcriptional response to hypoxia

**Fig. 4:**
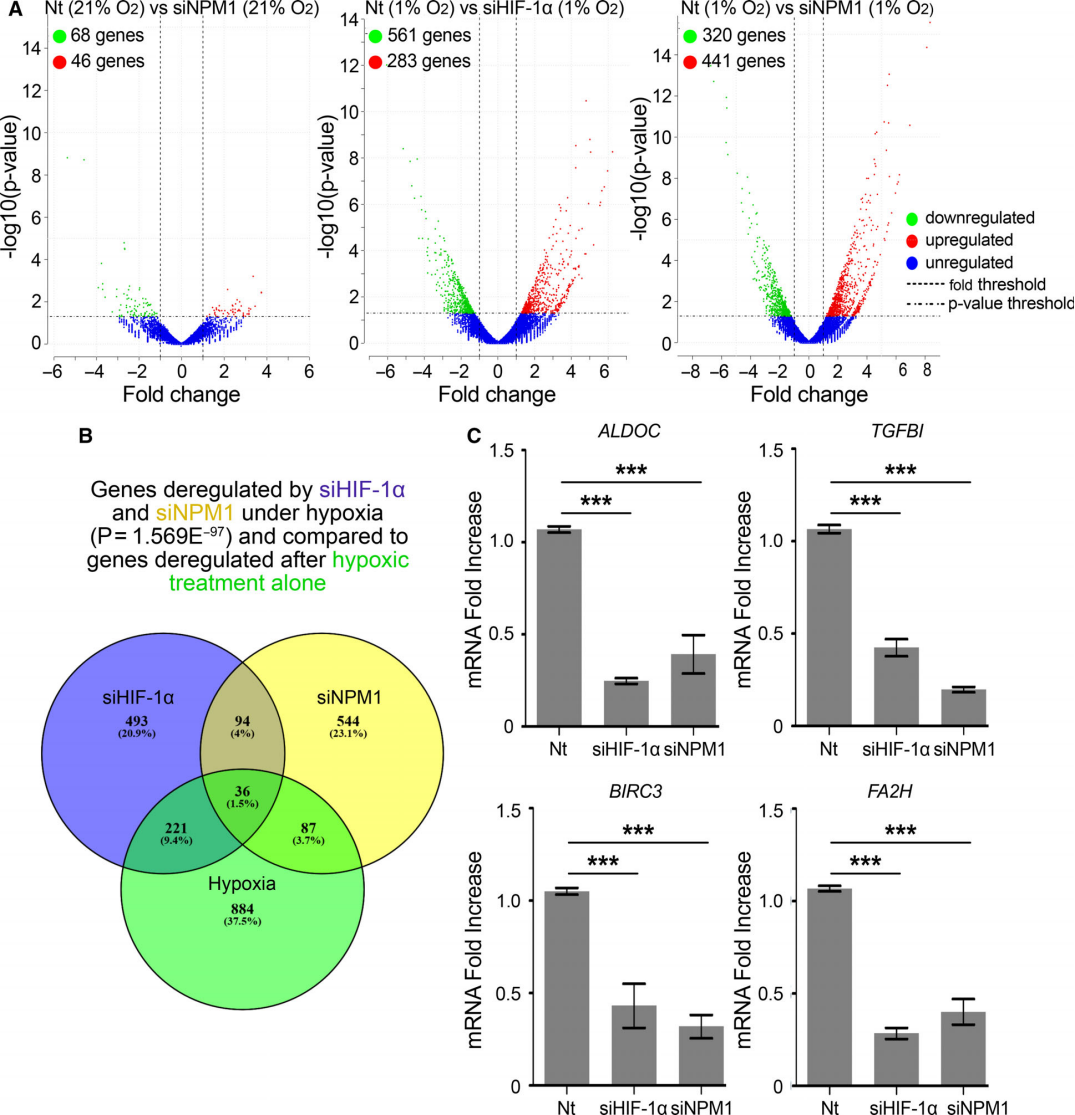
HIF‐1α and NPM1 coregulate a significant number of genes under hypoxia. HeLa cells treated with control (Nt) or NPM1 siRNA (siNPM1) for 24 h and incubated at 21% or 1% O_2_ for 24 h were then processed for 3′ mRNA sequencing. Values represent the mean of two independent experiments. (A) Volcano plots of genes showing significantly different expression levels after NPM1 (siNPM1) or HIF‐1α (siHIF‐1α) silencing compared to control (Nt) in cells under normoxia (21% O_2_) or hypoxia (1% O_2_). Normalized enrichment score from Gene Set Enrichment Analysis is shown. Statistical analysis was performed with DESeq through the Bioconductor package metaseqR [21]; *P*‐value < 0.05. (B) Venn diagram representing the number of genes significantly deregulated after HIF‐1α (Magenta) or NPM1 (Yellow) silencing under hypoxia in comparison to genes deregulated after hypoxic treatment alone (Green). (C) *ALDOC, TGFBI*, *BIRC3,* and *FA2H* expression levels were determined by RT‐PCR as indicated. Results are shown as fold decrease in relation to the respective control conditions (Nt) and represent the mean of two independent experiments performed in quadruplet ± SD (*n* = 8; ***P < 0.001; Statistical variance between two groups of values was calculated by two‐tailed Student's *t*‐test).

To address the above hypothesis, we performed sequencing of RNA extracted from HeLa cells subjected or not to NPM1 silencing, both under normoxia or hypoxia, or HIF‐1α silencing under hypoxia. In cells treated with control siRNAs (Nt), hypoxia heavily affected gene expression, with 1068 genes exhibiting altered mRNA levels (487 downregulated, 581 upregulated) when compared to normoxia (Fig. S8A). These genes are mostly involved in transcriptional regulation, response to hypoxia or drugs and control of apoptosis, angiogenesis, cell cycle, and metabolism (Fig. S8B). Knocking‐down NPM1 in normoxic cells resulted in the differential expression of a limited number of genes, 114 in total (68 down‐, 46 upregulated), which are mainly implicated in functions related to the immune response (Fig. 4A left panel; Fig. S8C,D) a subset of which (33 genes) were also regulated by HIF‐1 (Fig. S8C,E). In contrast, when NPM1 silencing was performed in hypoxic cells (Fig. 4A, right panel), it had a profound effect on gene expression with 761 deregulated genes (320 down‐, 441 upregulated), 123 of which were common with the ones affected by the hypoxic shift (Fig. 4B). A similar, strong, effect on differential gene expression was also observed when HIF‐1α was silenced under hypoxia with 844 deregulated genes (561 down‐, 283 upregulated; Fig. 4A, middle panel), 257 of which were common with the ones affected by hypoxia (as compared to normoxia; Fig. 4B). Analysis of those results revealed a significant number of genes commonly regulated by HIF‐1α and NPM1 (130 genes in total, out of which 36 also deregulated during the hypoxic shift; Fig. 4B). These common genes were involved in processes known to rely on HIF‐1 and hypoxia‐mediated reprogramming, such as cell adhesion, migration and ECM organization, redox and apoptosis control, metabolism, and angiogenesis (Fig. S8F). From the 67 genes that were upregulated by hypoxia and repressed by both NPM1 and HIF‐1α knockdown (Fig. S9), marker genes were selected as typical examples of NPM1/HIF‐1α‐dependent cellular functions (*ALDOC:* metabolism*, BIRC3:* apoptosis, *TGFBI:* angiogenesis and ECM organization*, FA2H:* oxidation‐reduction), for validation of the RNA‐sequencing results with RT‐PCR. Indeed, expression of *ALDOC, BIRC3, TGFBI*, and *FA2H* was decreased when either HIF‐1α or NPM1 were silenced under hypoxia (Fig. 4C). These data support the notion that NPM1, by stabilizing the interaction between HIF‐1 and HRE‐containing chromatin, supports the general transcriptional response to hypoxia, at least in cells (such as most cancer cells) in which ERK1/2 have been activated.

### 3.6 NPM1 and its association with HIF‐1 are necessary for cancer cell adaptation and survival under hypoxia

#### 3.6.1 NPM1 is required for HIF‐1‐dependent reprogramming of lipid metabolism under hypoxia

**Fig. 5:**
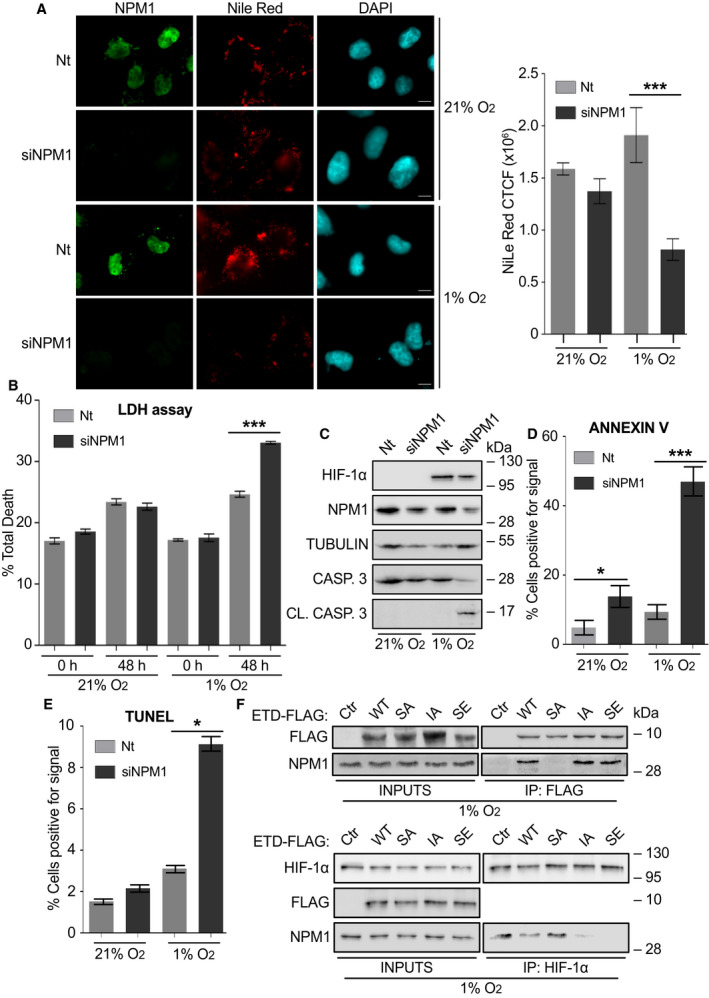
The NPM1/HIF‐1α interaction is essential for HIF‐1‐mediated metabolic adaptation and survival of cancer cell under hypoxia. (A) Left: Immunofluorescence microscopy images of HeLa cells treated with control (Nt) or NPM1 siRNA (siNPM1) for 24 h, incubated at 21% or 1% O_2_ for 24 h and then processed for detection of NPM1 (green) and lipid droplets (red). Nuclei are stained with DAPI (cyan; Scale bars: 10 μm). Right: Graph analysis of Nile Red fluorescent signal quantified with ImageJ software. Values represent corrected total cell fluorescence (CTCF) and are mean ±  SEM of measurements of 35 individual cells per condition (Statistical variance between two groups of values was calculated by two‐tailed Student's *t*‐test, ***P < 0.001). (B) Cell death analysis in HeLa cells transfected with control (Nt) or NPM1 siRNA (siNPM1) and, 24 h post‐transfection, incubated at 21% or 1% O_2_ for 48 h. Results are the mean of two independent experiments performed in quadruplets ±  SEM (Statistical variance between two groups of values was calculated by two‐tailed Student's *t*‐test, ***P < 0.001). (C) Immunoblotting analysis of lysates of HeLa cells transfected with control (Nt) or NPM1 siRNA (siNPM1) and incubated at 21% or 1% O_2_ for 24 h using antibodies against HIF‐1α, NPM1, tubulin, uncleaved (inactive) caspase 3 and cleaved (active) caspase 3 as indicated. Certain panels show single blot areas that correspond to the indicated molecular weight marker and were cut after blotting for analysis with different antibodies. (D) Quantification of Annexin V staining as detected by fluorescence microscopy of HeLa cells treated as in (C). Values are given as % ratio of Annexin V‐positive cells in relation to the total number of cells per condition ± SEM (*n* = 150 cells; Statistical variance between two groups of values was calculated by two‐tailed Student's *t*‐test, *P < 0.05; ***P < 0.001). (E) Quantification of TUNEL fluorescent signal in HeLa cells treated as in (C). Values are given as % ratio of fluorescence positive cells in relation to the total number cells ± SEM (*n* = 250 cells; Statistical variance between two groups of values was calculated by two‐tailed Student's *t*‐test, *P < 0.05). (F) Immunoblotting of soluble extracts (INPUTS) and anti‐FLAG (upper panels) or anti‐HIF‐1α (lower panels) IP of Huh7 cells grown at 1% O_2_ and treated with ˜380 nm of the indicated TAT‐ETD‐FLAG forms for 5 h, using antibodies against Flag, HIF‐1α, and NPM1 as indicated. Panels show single blot areas that correspond to the indicated molecular weight marker and were cut after blotting for analysis with different antibodies; images are representative of two independent experiments.

To validate the functional significance of our findings for the ability of cells to survive and proliferate under mild hypoxic conditions (1% O_2_), we tested various cellular functions known to depend on HIF‐1. We have previously shown that cancer cells respond to hypoxia by accumulating triacylglycerol in lipid droplets [8, 14]. In agreement with our transcriptional data (Fig. 3A–C), silencing of NPM1 significant decreased lipid droplet accumulation under hypoxia in HeLa cells, whereas there was no effect under normoxia (Fig. 5A), suggesting that NPM1 expression is important for metabolic adaptation to hypoxia.

#### 3.6.2 Depletion of NPM1 induces cell death by apoptosis under hypoxia

In addition to inhibition of metabolic reprogramming, silencing of NPM1 also increased cell death rate only under hypoxia (Fig. 5B) and stimulated apoptosis as evidenced by activation and cleavage of caspase 3 (Fig. 5C), loss of membrane asymmetry (Annexin V staining; Fig. 5D, Fig. S10A) and fragmentation of DNA (TUNEL assay; Fig. 5E, Fig. S10B) in HeLa cells. In all these processes, the effects of NPM1 silencing were visible or exacerbated only after incubation for 24 h or more under hypoxia. Therefore, lack of NPM1 has little effect on survival/proliferation of cells grown under normoxic conditions, but becomes deleterious under low oxygen conditions, due to suboptimal activity of HIF‐1 and curtailed adaptive transcriptional response.

#### 3.6.3 Apoptosis‐inducing cell‐penetrating ETD‐based peptides disrupt the HIF‐1α/NPM1 complex

A question remaining is whether NPM1 depletion does not only affect HIF‐1‐depedent processes but also has unspecified negative effects unrelated to HIF‐1. In a recent study [11], we could show that inclusion of cell‐penetrating peptides, comprising the ETD region of HIF‐1α in various mutant forms, in the culture medium of three different types of cancer cells could drastically reduce HIF‐1 activity (without affecting HIF‐2) and inhibit proliferation as well as migratory and colony formation abilities of the cells and trigger apoptotic death only under hypoxic conditions. In view of the results reported herein, we tested whether the same peptides could affect the HIF‐1α/NPM1association. Introduction of these TAT‐ETD‐FLAG peptides into hypoxic Huh7 cells followed by their IP showed that peptides that contain either sites for ERK1/2 phosphorylation (WT, IA) or a phosphomimetic mutation (SE), bound efficiently to endogenous NPM1, while the phospho‐deficient SA form displayed no interaction (Fig. 5F; upper panels), starkly reproducing the *in vitro* binding data shown in Fig. 1. Under the same conditions, IP of HIF‐1α showed that HIF‐1α/NPM1 association could be disrupted by the NPM1‐binding ETD peptides (Fig. 5F; lower panels). These results strongly suggest that it is not just the presence of NPM1 but rather its ability to interact with ERK1/2‐modified HIF‐1α that supports the transcriptional function of HIF‐1 and cancer cell adaptation to hypoxia.

### 3.7 Expression of NPM1 and NPM1/HIF‐1 co‐upregulated genes is increased in human cancers and correlates with hypoxic tumor status and bad patient prognosis

#### 3.7.1 Expression of NPM1 correlates with HIF‐1 and is a bad prognostic factor in different cancer types

**Fig. 6:**
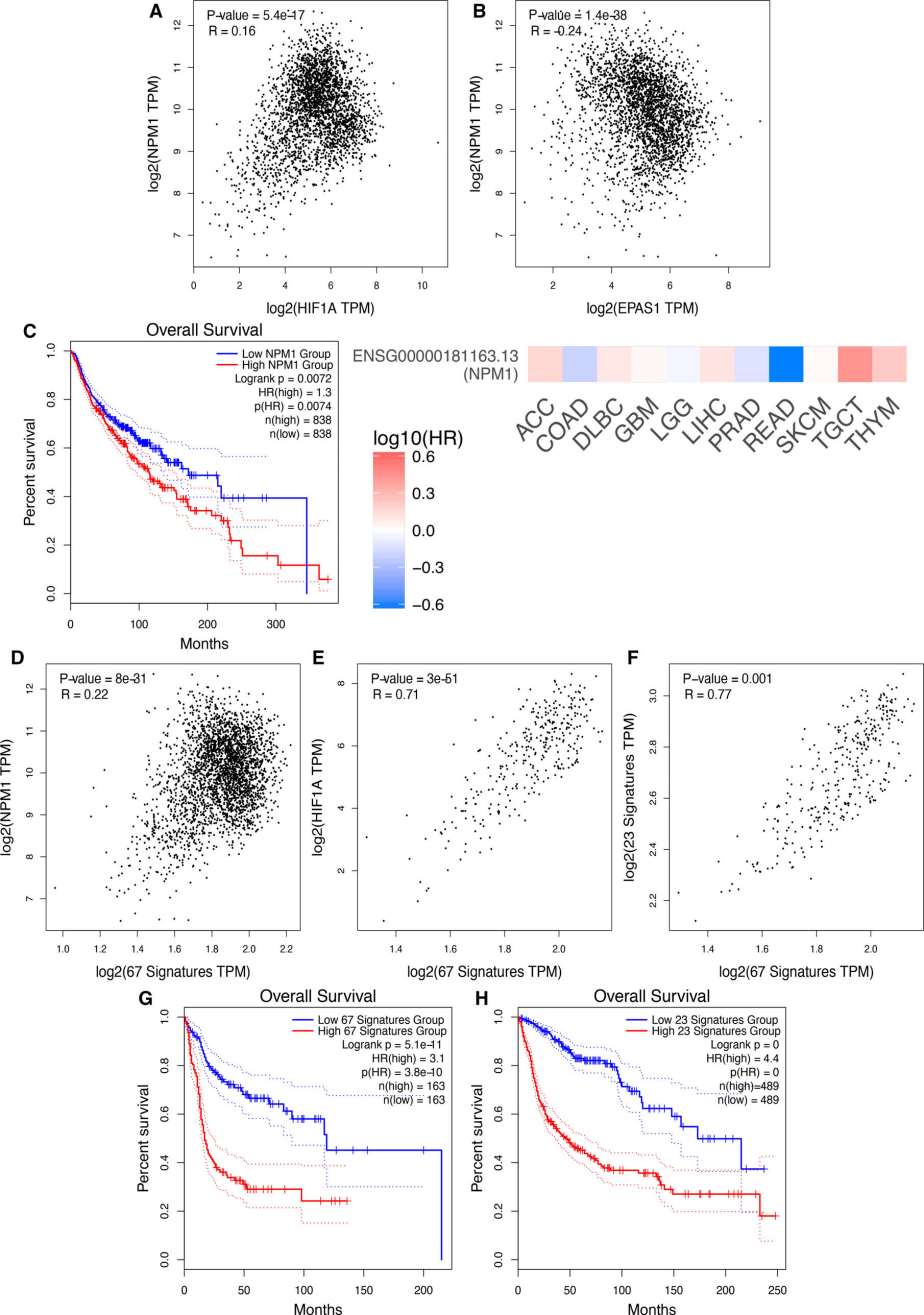
Expression of *NPM1* and NPM1/HIF‐1 co‐upregulated genes in human cancers correlated with hypoxic status and patient prognosis. (A, B) Correlation between the expression of *NPM1* and the expression of either *HIF1A* (A) or *EPAS1* (B) in the combined samples of the 11 cancer types shown in Fig. S11B. (C) Left: Kaplan–Meier overall survival curves for patients corresponding to the 11 human cancer types shown in Fig. S11B and assigned to high (Red) or low (Blue) *NPM1* expression in their tumor samples. Right: Heatmap showing the prognostic impact of *NPM1* expression in each of the 11 cancer types as indicated. Hazard ratios (HR; Color key) are in logarithmic scale (log_10_); Red and blue squares signify higher and lower risks, respectively. (D–F) Correlation between the expression *NPM1* (D) or *HIF1A* (E) or a hypoxia 23‐gene signature (F) and the signature of the 67 genes commonly upregulated by HIF‐1 and NPM1 under hypoxia in HeLa cells (this study) in the combined samples of three human cancer types (DLBC, GBM and THYM). (G, H) Kaplan–Meier overall survival curves for patients corresponding to three human cancer types (DLBC, GBM and THYM) and assigned to high (Red) or low (Blue) expression of the 67 genes upregulated by both HIF‐1 and NPM1 in Hela cells (this study) (G) or expression a 23‐hypoxia gene signature (H). Spearman correlation analysis in A, B, D, E, F and overall survival analysis in C, G, H were analyzed by GEPIA2 visualization and evaluation platform as reported in [23]. Continuous red/blue lines in C, G, H represent mean values whereas dotted lines are indicative of variation.

In order to examine whether the phosphorylation‐dependent interaction between HIF‐1α and NPM1 may play a role in human patient tumor growth, we analyzed *NPM1* expression as well as our RNA‐seq datasets in the context of the publicly available gene expression data from TCGA and GTEx using the GEPIA2 web‐based platform [23]. NPM1, much like HIF‐1, expression has been long known to be increased in certain human tumors [29]. Indeed, the data mining process with GEPIA2 for analysis of *NPM1* mRNA levels in 33 different human cancer types (Table S6) revealed that *NPM1* expression is significantly higher in 11 types of human tumors in comparison to paired normal tissues (Fig. S11A,B). In these 11 cancer types, *NPM1* expression is positively correlated with expression of *HIF1A* (Fig. 6A) but not with *EPAS1* (Fig. 6B) which encodes HIF‐2α, in line with our data showing that NPM1 interacts only with the HIF‐1α isoform (Fig. 2D). Interestingly, high *NPM1* expression is associated with negative prognostic outcome in the combined cohort of patients with these 11 cancer types (Fig. 6C, left panel) and with higher risk in seven of them when analyzed individually (Fig. 6C, right panel).

#### 3.7.2 Expression of HIF‐1/NPM1 co‐upregulated genes correlates with a hypoxic signature and poor patient prognosis in three different cancer types

To then test the relationship between NPM1 and HIF‐1α in the high NPM1 cancer types, we analyzed the expression of the 67 genes commonly upregulated by NPM1 and HIF‐1α under hypoxia (Fig. S9). The 67 NPM1/HIF‐1α‐dependent gene signature was highly expressed in three cancer types with elevated NPM1 expression, namely lymphoid neoplasm diffuse large B‐cell lymphoma (DLBC), glioblastoma multiforme (GBM), and thymoma (THYM; Fig. S11C). To analyze the expression of known hypoxia target genes, we created a hypoxia gene signature, which comprised a subset of 23 genes with the strongest functional correlation with HIF‐1 (Fig. S12A; Table S7). Based on the expression of this hypoxic gene signature, five out of the 11 cancer types with high NPM1 could be characterized as ‘hypoxic’ (Fig. S12B). Moreover, three of the ‘hypoxic’ cancer types were the same as the ones exhibiting significantly high expression levels of the 67 NPM1/HIF‐1α‐dependent gene signature (namely, DLBC, GBM, and THYM; Fig. S11C). Additionally, in these three cancer types not only *NPM1* (Fig. 6D) and *HIF1A* (Fig. 6E) expression but also the expression of the hypoxic gene signature (Fig. 6F) positively correlated with the expression of the 67 genes commonly upregulated by HIF‐1α and NPM1. Remarkably, both the 67 NPM1/HIF‐1α‐dependent gene signature gene and the hypoxic gene signatures were associated with poor prognostic outcome in the cohort of patients with DLBC, GBM, or THYM cancer types (Fig. 6G,H). These cancer patient data support the notion that the NPM1/HIF‐1α interaction also occurs in solid tumors and it is highly involved in the response of cancer cells to the hypoxic tumor microenvironment, which in turn can facilitate tumor growth and resistance to therapy.

## 4 Discussion

### 4.1 NPM1, a novel isoform‐specific HIF‐1 interacting partner, is essential for the transcriptional response and cellular adaptation to hypoxia

**Fig. 7:**
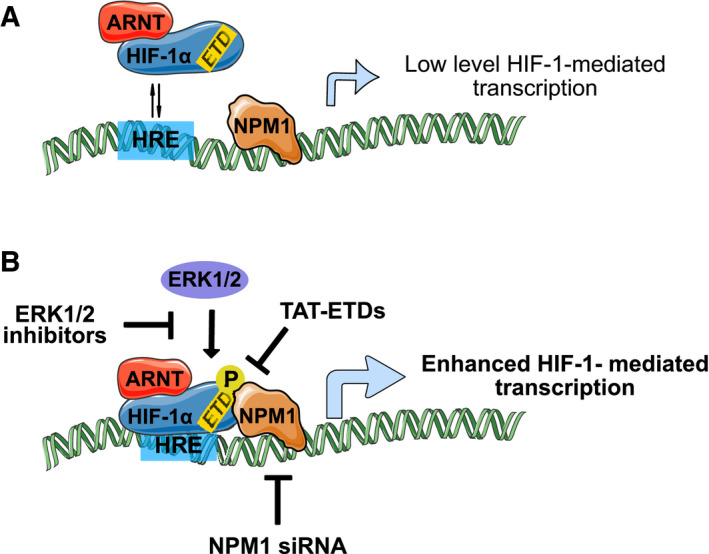
A model for the role of the HIF‐1/NPM1 complex during hypoxia. NPM1 marks and may organize hypoxia‐inducible promoters. (A) In the absence of ERK‐mediated phosphorylation (e.g., in quiescent cells), HIF‐1α binding to NPM1 is weak resulting in unstable association of HIF‐1 with a neighboring HRE and basal levels of transcriptional activity. (Β) In cells with elevated ERK1/2 activity (e.g., in rapidly proliferating and growing cells, such as cancer cells), HIF‐1α is directly phosphorylated by ERK1/2 at the ETD, which can then bind strongly to NPM1 and mediate stable association of HIF‐1 with a neighboring HRE resulting in maximal activation of transcription. Depletion of NPM1 (e.g., by silencing) or inhibition of HIF‐1α phosphorylation (e.g., by mutation of the ERK sites or ERK inhibitors) or disruption of HIF‐1α/NPM1 association (e.g., by cell‐penetrating ETD peptides) inhibits HIF‐1‐mediated transcription and trigger apoptosis of cancer cells under hypoxia.

Intrigued by the fact that phosphorylation of the HIF‐1α ETD by ERK1/2 limits the intranuclear mobility of HIF‐1α in living cells, we used an unbiased proteomic approach to screen for ETD‐interacting nuclear proteins. This led to the identification of NPM1 as a direct binding partner of HIF‐1α both *in vitro* and inside cancer cells. Our functional data suggest that the HIF‐1α/NPM1 interaction forms the basis of a regulatory mechanism that connects the status of ERK activation (and of, correspondingly, cellular proliferation) with the level of HIF‐1 transcriptional activity and the ability of cells to respond and adapt to hypoxia (Fig. 7). Furthermore, they shine further light on the means by which HIF‐1 selects, associates with, and activates hypoxia target gene promoters and how these may differ from the corresponding HIF‐2‐dependent processes. These hypotheses are supported by our experimental results showing that (a) direct binding of NPM1 to the HIF‐1α ETD (residues 616–658) is strengthened when ETD can be phosphorylated by ERK or contains a phosphomimetic mutation at the ERK sites; (b) NPM1 interacts with both the HIF‐1α/ARNT heterodimer and HRE‐containing chromatin but shows no association with HIF‐2α; (c) depletion of NPM1 or inhibition of its interaction with HIF‐1α destabilizes the association of HIF‐1 with functional HREs or components of active chromatin and reduces drastically its transcriptional activity; (d) a significantly large number of genes are commonly regulated by HIF‐1 and NPM1 under hypoxia; and (e) analysis of publicly available cancer patient data reveals a strong association between hypoxia, NPM1, and the HIF‐1/NPM1 co‐dependent gene expression and exposes their correlation with bad patient prognosis.

NPM1, the interaction partner of HIF‐1α identified in the present study, is an abundant phosphoprotein containing independent but partially overlapping functional domains which may facilitate interactions with chromatin components, transcription factors, or nucleic acids [32, 33]. Moreover, NPM1 is a histone chaperone, a function that depends on its phosphorylation, p300‐mediated acetylation, and oligomerization status, and association of NPM1 with components of the transcription machinery has been suggested to enhance chromatin transcription [34, 35]. Our results now demonstrate that the transcriptional response to hypoxia requires the presence of NPM1 and its direct interaction with HIF‐1α, which involves the C‐terminal 37 amino acid aromatic stretch of NPM1 present only in the major B23.1 splicing variant. Interestingly, the same C‐terminal stretch was recently found to be essential for NPM1 binding to the *PD‐L1* gene promoter and for increased expression of PD‐L1 in triple‐negative breast cancer [36]. Moreover, mutations in this area are responsible for the cytoplasmic mislocalization and malfunction of NPM1 during Acute Myelogenous Leukemia [37], showing that this small C‐terminal domain of NPM1 mediates important interactions. The NPM1 C‐terminal basic region has also been shown to interact with the NF‐κB p65 subunit and enhance its DNA binding [38]. Unlike the large number of hypoxia‐related genes affected by depletion of NPM1 under hypoxia, the small number of genes deregulated by NPM1 silencing under normoxia was mainly involved in the immune response. This is in agreement with the involvement of NPM1 in NF‐κB activation and may point to an additional connection between inflammation‐ and hypoxia‐induced transcriptional responses. Given that in response to various types of stress, NPM1 has also been shown to translocate from the nucleolus to the nucleoplasm, where it can associate with and affect stress‐related transcription factors [39], it can also be speculated that NPM1 marks promoter regions of genes that need to be rapidly activated in response to external stimuli such as oxygen concentration or pro‐inflammatory agents. NPM1 has demonstrated ability to undergo homotypic and heterotypic liquid–liquid phase separation (LLPS), which may be critical for its nucleolar function [27, 40]. As phase separation condensates may also be involved in RNA Pol II‐mediated transcription [41], LLPS may also play a role in the involvement of NPM1 in the regulation of mRNA synthesis. Our data have shown that the interaction with HIF1α requires the very C‐terminal aromatic and globular domain of NPM1 and not its intrinsically disordered acidic and basic regions that mediate LLPS, making the possibility that the NPM1/ HIF‐1α interaction occurs in the context of LLPS very unlikely. However, the LLPS forming properties of NPM1 may be important for concentrating other essential transcription coactivators at open and rapidly activated gene promoters, such as those regulated by hypoxia. Indeed, it has been shown that most HIF‐1 target genes reside in open chromatin loci with bound but paused RNA polymerase II (RNAPII) and display basal transcriptional activity even under normoxia [42, 43, 44]. Under hypoxia, elongation by RNAPII is triggered when HIF‐1 recruits the CDK‐8‐Mediator and super elongation (SEC) complexes [43]. Additional transcriptional coactivators of HIF‐1 target genes include the histone acetyltransferases CBP/p300 [45], the binding of which to HIF‐1α also involves STAT3 [46], the chromatin‐remodeling SWI/SNF complex [47, 48, 49], and the pyruvate kinase isoform PKM2, which, like NPM1, is a HIF‐1 target gene [50]. However, chromatin modification/remodeling and transcription elongation in response to hypoxia need to be preceded by stable binding of HIF‐1 to the HREs via its N‐terminal DNA‐binding domains. We propose that this is secured by NPM1, by preoccupying functional HRE‐containing promoters already under normoxia and providing additional stabilizing anchorage via binding to the ETD of HIF‐1α, after its hypoxia‐triggered induction and subsequent nuclear import and heterodimerization with ARNT. Furthermore, the HIF‐1α/NPM1 interaction can serve more purposes: it provides an additional level of regulation by the signaling pathways leading to ERK activation, it establishes a positive feed‐forward mechanism by HIF‐1‐mediated stimulation of NPM1 expression and it facilitates transcriptional activation by the histone chaperone and chromatin‐remodeling abilities of NPM1. The HIF‐1α/NPM1 interaction, mediated by a domain not conserved between HIF‐α isoforms, may also be one of the elements that differentiates gene targets between HIF‐1 and HIF‐2. It is well established that while both HIFs recognize the same core HRE‐motif they can affect the transcription of distinct set of genes [51]. Furthermore, several studies have shown that HIF‐2 is prone to bind to DNA elements distal from the transcription start sites, while HIF‐1 is more often found bound to promoter regions proximal to transcription start sites [51, 52, 53]. Given that NPM1 associates with transcriptionally active RNAPII and shows nucleosome disassembly activity at transcription start sites [34, 35], its preference for HIF‐1α may act as a marker for favored HIF‐1 binding to HREs proximal to transcription start sites.

### 4.2 The NPM1/HIF‐1α association provides an ERK‐controlled switch that can be targeted in cancer cells

The control of the HIF‐1α/NPM1 interaction by ERK‐dependent phosphorylation of HIF‐1α extends the role played by the ERK pathway both in normal and cancer cells. ERK pathway activation involves not only stimuli such as growth factors, mitogens, and cytokines but also environmental conditions like hypoxia. Its activation is also correlated with carcinogenesis since it is required for high proliferation rates, cell growth, cell survival, and evasion of apoptosis [54]. Active ERKs could therefore also lead to a more tumor‐promoting phenotype via the HIF‐1/NPM1 axis in the hypoxic microenvironment of solid tumors. This is in line with the pro‐tumorigenic roles attributed individually to both NPM1 and HIF‐1. As already mentioned, NPM1 expression is increased in various cancer types and it has been associated with progression to more advanced stages of disease. Its oncogenic potential is manifested through both increased proliferation and inhibition of apoptosis [29]. In our cell culture experiments, depletion of NPM1 had an insignificant or very mild effect on cell death and apoptosis under normoxia but apoptosis was dramatically stimulated when NPM1 depletion was combined with hypoxia. This agrees with the previously suggested indirect antiapoptotic role of NPM1 [29] and, taking into account our data, it may also be proposed that NPM1 protects from apoptosis by increasing, through HIF‐1, the expression of antiapoptotic genes, such as BIRC3 which was tested in our study. Support for this idea also comes from our RNA‐seq results and our analysis of cancer patient data. The former revealed a significant number of genes that depend on both HIF‐1 and NPM1 for expression under hypoxia and negatively regulate apoptosis. The latter demonstrated the elevated expression of NPM1 in several different human cancer types, its association with bad prognosis, and its correlation with *HIF1A* but not *EPAS1* (encoding HIF‐2α) expression in full support of our *in vitro* cell data. Furthermore, a signature of the 67 genes found from our analysis to be commonly upregulated by HIF‐1 and NPM1 was significantly higher in at least three different human tumor types (DLBC, GBM, THYM) and correlated with an independent hypoxic signature and negative prognostic outcome.

The demonstration of the interaction between HIF‐1, an established target for anticancer strategies [2], and NPM1, also associated with carcinogenesis, raises the interesting possibility that targeting this interaction could serve as an efficient means to curtail HIF‐1 activity and decrease cancer cell survival in hypoxic tumors. The fact that the cell permeable TAT‐ETD‐FLAG peptides, which, as we have previously shown, specifically inhibit HIF‐1 activity and trigger apoptosis in different cancer cell lines [11], efficiently disrupt the HIF‐1α/NPM1 association provides proof‐of‐principle that remains to be tested as a therapeutic intervention *in vivo*.

## 5 Conclusion

We have shown that ERK‐mediated phosphorylation of HIF‐1α controls its association with chromatin through its physical interaction with NPM1, a histone chaperone, and chromatin remodeler. This interaction allows HIF‐1 to select and stably bind to HRE‐containing promoters, making NPM1 an essential coactivator of hypoxia target genes, a role also supported by analysis of cancer patient data. Depletion of NPM1 or disruption of its association with HIF‐1α selectively curtails the ability of cancer cells to survive under low oxygen. Targeting the HIF‐1α/NPM1 interaction in hypoxic tumors may therefore form the basis of a novel anticancer strategy.

## Conflict of interest

The authors declare no conflicts of interest.

## Author contributions

KK, AK: performed experiments, AG: transcriptomic analysis, MS and GP: mass spectrometry, GS and IM: design, supervision, funding, and paper writing.

### Peer Review

The peer review history for this article is available at https://publons.com/publon/10.1002/1878‐0261.13080.

## Supporting information


**Fig. S1**. Schematic representation of HIF‐1α forms used in this study.
**Fig. S2**. HIF‐1α phosphorylation by ERK1/2 stimulates HIF‐1α binding to chromatin components.
**Fig. S3**. HIF‐1α phosphorylation by ERK1/2 stimulates HIF‐1α binding to chromatin components.
**Fig. S4**. SDS/PAGE analysis of recombinant HIF‐1α and NPM1 forms used in this study.
**Fig. S5**. NPM1 immunofluorescence under normoxic conditions.
**Fig. S6**. NPM1 is essential for HIF‐1 transcriptional activity and stable binding to HRE and components of open chromatin.
**Fig. S7**. NPM1 occupies functional HRE sequences.
**Fig. S8**. HIF‐1α and NPM1 co‐regulate a significant number of genes under hypoxia.
**Fig. S9**. The 67 genes co‐upregulated by NPM1 and HIF‐1α under hypoxia.
**Fig. S10**. Annexin V and TUNEL staining in HeLa cells.
**Fig. S11**. Expression of NPM1 and a 67 gene‐signature co‐upregulated by NPM1 and HIF‐1α in various cancer types.
**Fig. S12**. STRING analysis of GSEA dataset and expression of a 23‐gene hypoxic signature in various cancer types.
**Table S1**. List of non‐target and specific siRNAs used in this study.
**Table S2**. List of antibodies and working dilutions used in this study.
**Table S3**. List of DNA primers for RT‐PCR and CHIP analysis used in this study.
**Table S4**. Measured parameter estimates of FRAP experiments.
**Table S5**. Peptide identification details from mass spectrometry.
**Table S6**. Tumor type abbreviations.
**Table S7**. The 23‐gene hypoxia signature after STRING analysis from GSEA original dataset.

## Data Availability

The data discussed in this publication have been deposited in NCBI's Gene Expression Omnibus [55] and will be accessible after publication through GEO Series accession number GSE158890 (https://www.ncbi.nlm.nih.gov/geo/query/acc.cgi?acc=GSE158890). Requests for materials should be addressed to GS and IM.
