# Supplementary material for: ERK signaling controls productive HIF‐1 binding to chromatin and cancer cell adaptation to hypoxia through HIF‐1α interaction with NPM1
Source: Mol Oncol. 2021 Sep 9;15(12):3468–89. doi: 10.1002/1878-0261.13080 (PMC8637566; doi:10.1002/1878-0261.13080)
Supplement: Supplementary file 1 — Fig. S1. Schematic representation of HIF‐1α forms used in this study. Fig. S2. HIF‐1α phosphorylation by ERK1/2 stimulates HIF‐1α binding to chromatin components. Fig. S3. HIF‐1α phosphorylation by ERK1/2 stimulates HIF‐1α binding to chromatin components. Fig. S4. SDS/PAGE analysis of recombinant HIF‐1α and NPM1 forms used in this study. Fig. S5. NPM1 immunofluorescence under normoxic conditions. Fig. S6. NPM1 is essential for HIF‐1 transcriptional activity and stable binding to HRE and components of open chromatin. Fig. S7. NPM1 occupies functional HRE sequences. Fig. S8. HIF‐1α and NPM1 co‐regulate a significant number of genes under hypoxia. Fig. S9. The 67 genes co‐upregulated by NPM1 and HIF‐1α under hypoxia. Fig. S10. Annexin V and TUNEL staining in HeLa cells. Fig. S11. Expression of NPM1 and a 67 gene‐signature co‐upregulated by NPM1 and HIF‐1α in various cancer types. Fig. S12. STRING analysis of GSEA dataset and expression of a 23‐gene hypoxic signature in various cancer types. Table S1. List of non‐target and specific siRNAs used in this study. Table S2. List of antibodies and working dilutions used in this study. Table S3. List of DNA primers for RT‐PCR and CHIP analysis used in this study. Table S4. Measured parameter estimates of FRAP experiments. Table S5. Peptide identification details from mass spectrometry. Table S6. Tumor type abbreviations. Table S7. The 23‐gene hypoxia signature after STRING analysis from GSEA original dataset. [file MOL2-15-3468-s001.pdf]

Supplementary Information

ERK signaling controls productive HIF-1 binding to chromatin and cancer cell adaptation to hypoxia through HIF-1α interaction with NPM1

Kreon Koukoulas[1], Antonis Giakountis[2,3], Angeliki Karagiota[1], Martina Samiotaki[3], George Panayotou[3], George Simos[1,2] and Ilias Mylonis[1].

[1] Laboratory of Biochemistry, Faculty of Medicine and [2] Department of Biochemistry and Biotechnology, University of Thessaly, Biopolis, Larissa, 41500, Greece.
[3] Institute for Bio-innovation, BSRC "Alexander Fleming", Vari, 16672, Greece.
[4] Gerald Bronfman Department of Oncology, Faculty of Medicine, McGill University, Montreal, H4A 3T2, Canada.

Correspondence to : George Simos or Ilias Mylonis
Email: simos@med.uth.gr or mylonis@med.uth.gr

Supplementary Information Includes:

Supplementary Table S1, related to Methods section
Supplementary Table S2, related to Methods section
Supplementary Table S3, related to Methods section
Supplementary Table S4, related to Figure 1
Supplementary Table S5, related to Figure 1
Supplementary Table S6, related to Figure 6 and Supplementary Figures S11-S12
Supplementary Table S7, related to Figure 6
Supplementary Figure S1, related to Methods section and Figures 1, 2, 5
Supplementary Figure S2, related to Figure 1
Supplementary Figure S3, related to Figure 1
Supplementary Figure S4, related to Figure 1
Supplementary Figure S5, related to Figure 2
Supplementary Figure S6, related to Figure 3
Supplementary Figure S7, related to Figure 3
Supplementary Figure S8, related to Figure 4
Supplementary Figure S9, related to Figure 4
Supplementary Figure S10, related to Figure 5
Supplementary Figure S11, related to Figure 6
Supplementary Figure S12, related to Figure 6

Supplementary Table S1. List of non-target and specific siRNAs used in this study.

siRNA	Sequence (5’-3’)	Reference
AllStars Non target siRNA	Propriatary	Qiagen (cat. no. 1027280)
HIF-1α HP siRNA	AGGAAGAACTATGAACATAAA	Qiagen (cat. no. SI02664053)
NPM1 Predesign Chimera RNAi	Propriatary	Abnova (cat. no. H00004869_R01)
NPM1 FlexiTube siRNA	AAAGGTGGTTCTCTTCCCAAA	Qiagen (cat. no. SI2654960)

Supplementary Table S2. List of antibodies and working dilutions used in this study.

PRIMARY ANTIBODIES

Antibody	Reference	Working dilution
Rabbit polyclonal anti-HIF-1α	Lyberopoulou et al., 2007	Western Blot 1:2000, Immunoprecipitation and CHIP 1μg/mg of total protein extract
Mouse monoclonal anti-HIF-1α	BD Transduction Laboratories, 610959	IF 1:500
Rabbit polyclonal anti-NPM1	Millipore, ABS667	Western Blot 1:2000, IF 1:1000, Immunoprecipitation and CHIP 1μg/mg of total protein extract
Mouse monoclonal anti-NPM1	Millipore, MAB937	Western Blot 1:3000
Rabbit polyclonal anti-HIF-2α	Novus Biologicals, NB100-122	Western Blot 1:1000
Mouse monoclonal anti-ARNT	BD Transduction Laboratories, 611079	Western Blot 1:500
Rabbit polyclonal anti-p44/42 MAPK	Cell Signaling, 9102S	Western Blot 1:1000
Rabbit polyclonal anti-Phospho-p44/42 MAPK	Cell Signaling, 9101S	Western Blot 1:1000
Rabbit polyclonal anti-Lipin1	Grimsey et al., 2008	Western Blot 1:1000
Rabbit polyclonal anti-Caspase 3	Cell Signaling, 9662S	Western Blot 1:1000
Rabbit polyclonal anti-Cleaved-Caspase 3	Cell Signaling, 9664S	Western Blot 1:1000
Mouse monoclonal anti-β-Actin	Cell Signaling, 3700S	Western Blot 1:5000
Mouse monoclonal anti-α-tubulin	Cell Signaling, 3873S	Western Blot 1:10000
Mouse monoclonal anti-FLAG M5	Sigma-Aldrich, F4042	Western Blot 1:10000, Immunoprecipitation 1μg/mg of total protein extract
Rabbit polyclonal anti-GFP	Thermo Fisher Scientific, A6455	Western Blot 1:2000

Rabbit polyclonal anti-GFP serum	Dr.H.Boleti (Hellenic Pasteur Institute, Athens, Greece)	Western Blot 1:2000, Immunoprecipitation 1µg/mg of total extract
anti-GFP single domain antibody (sdAb) conjugate to agarose beads (GFP-Trap)	ChromoTek Gmbh, gta-10	Immunoprecipitation 20µl slurry/mg of total protein extract
Mouse monoclonal anti-GST	Ximbio, 15TF2-1D10	Western Blot 1:10000
Mouse monoclonal anti-Acetyl Histone H4 (K5, K8,K12 or16)	Ximbio, 3HH4-2C2	Western Blot 1:1000
Rabbit IgG	Sigma, I5006-10	CHIP 1µg/mg of total protein extract

SECONDARY ANTIBODIES

Antibody	Reference	Working dilution
Goat Anti-Rabbit HRP	Cell Signaling, 7074	Western Blot 1:2000
Horse Anti-Mouse HRP	Cell Signaling, 7076	Western Blot 1:3000
Donkey Anti-Mouse IgG (FITCH)	Jackson ImmunoResearch, 715-095-151	IF 1:500
Donkey Anti-Rabbit IgG (Cy3)	Jackson ImmunoResearch, 711-165-152	IF 1:500

Supplementary Table S3. List of DNA primers for RT-PCR and CHIP analysis used in this study.

Oligonucleotide Name	Sequence (5’-3’)
DNA primers for RT-PCR	
hLPIN1+141-F	TTTCCACGTCCGCTTTGGG
hLPIN1+314-R	GTGGCCAGGTGCATAGGG
hsP4HA1-F	AGGGGTTGCTGTGGATTACC
hsP4HA1-R	GTCATGTACTGTAGCTCGGC
hsACTIN-F	CCAACCGCGAGAAGATGA
hsACTIN-R	CCAGAGGCGTACAGGGATAG
ALDOC-F	CTGCCACTGAGGAGTTCATC
ALDOC-R	CTCCACCATCTTCTCCACTG
FA2H-F	AACGAGCCTGTAGCCCTTGA
FA2H-R	ACTGCCACCGTGTACTCTGTT
TGFBI-F	GTCCACAGCCATTGACCTTT
TGFBI-R	ACCGCTCACTTCCAGAGAGA
BIRC3-F	CTTTGCCTGTGGTGGAAAAT
BIRC3-R	ACTTGCAAGCTGCTCAGGAT
DNA primers for CHIP analysis	
hLPIN1CHIPF1 (-2916 to -2686)	TGGGATCCTTTCTGCCCGGG
hLPIN1CHIPR1 (-2916 to -2686)	CACTGCTGAGCCCAGCTGGT
AGPAT2 HRE 5-6 (-1013 to -778) F	AAAAAGAGGGGCCGTGCTC
AGPAT2 HRE 5-6 (-1013 to -778) R	GTTCACATCCGCTTGGCAG
AGPAT2 HRE 4 (-685 to -492) F	AGACACACGCCCCAGTTG
AGPAT2 HRE 4 (-685 to -492) R	CAGAACCACAGCTCCCCAAG
AGPAT2 HRE 1-3 (-330 to -140) F	GTAACCTGGCAGAAGGCTGT
AGPAT2 HRE 1-3 (-330 to -140) R	CAGGGAAGGGCTAGGTGC
HSPB HRE F	GTTCCAGATGAGGGCTGAAC
HSPB HRE R	TCTGGACGTCTGCTCAGAAA
HSPBneg F	CTCAAACGGGTCATTG
HSPBneg R	TCGGCTGCGCTTTTTAT
HAMP F	CACATCTCAAGGGTCTGACAC
HAMP R	ATGAGCAGAATCAAGGTTCC
CASP9 F	GTGACGCAAGAGCGAATCCTT
CASP9 R	CAGGGCCAAGCCTCCCAT

Supplementary Table S4. Measured parameter estimates of FRAP analysis of different GFP-HIF-1α (upper panel) or GFP-ETD (lower pannel) peptide forms. GFF-NLS is used as a completely free and diffusible nuclear control. Parameters displayed are diffusion coefficient (D_{eff}), half-maximal recovery time ($t_{1/2}$) and mobile fraction (f_{mob}) (±s.d.). N: number of cells analyzed for each construct in two independent experiments.

**GFP-HIF-1α forms**	**D_{eff} (μm^2 s^{-1})**	**$t_{1/2}$ (s)**	**f_{mob}**	**N**
GFP-NLS	5.20 ± 0.75	0.08 ± 0.01	0.99 ± 0.01	15
GFP-HIF-1α WT	1.43 ± 0.44	0.30 ± 0.09	0.98 ± 0.02	21
GFP-HIF-1α +Kae	3.14 ± 1.35	0.17 ± 0.10	0.97 ± 0.02	15
GFP-HIF-1α IA	1.53 ± 0.44	0.29 ± 0.12	0.98 ± 0.03	21
GFP-HIF-1α IA/SA	3.55 ± 1.02	0.17 ± 0.07	0.99 ± 0.01	20
GFP-HIF-1α SE	0.75 ± 0.42	0.88 ± 0.37	0.87 ± 0.13	22

**GFP-ETD peptide forms**	**D_{eff} (μm^2 s^{-1})**	**$t_{1/2}$ (s)**	**f_{mob}**	**N**
GFP-NLS	5.20 ± 0.75	0.08 ± 0.01	0.99 ± 0.01	15
GFP-ETD WT	4.73 ± 1.20	0.09 ± 0.02	0.99 ± 0.02	20
GFP-ETD IA	4.70 ± 0.77	0.09 ± 0.01	1.00 ± 0.01	23
GFP-ETD IA/SA	4.65 ± 0.68	0.09 ± 0.02	1.00 ± 0.01	21
GFP-ETD SE	3.79 ± 1.75	0.14 ± 0.05	0.97 ± 0.04	21

Supplementary Table S5. Peptide identification details (sequence, Xcorr values, charge state and MH+ [Da]) of the protein band detected in association with ETD-SE (Fig. 1C). Five peptides were identified with confidence as parts of the protein nucleophosmin (NPM1; P06748).

Sequence	XCorr	Charge	MH+ [Da]	ΔM [ppm]	RT [min]
MSVQPTVSLGGFEITPPVVLR	4.12	2	2227.21807	1.10	110.85
VDNDENEHQLSLR	3.60	2	1568.73076	0.48	60.84
DELHIVEAEAMNYEGSPIK	3.47	3	2145.01828	0.62	98.22
MTDQEAIQDLWQWR	2.75	2	1819.84648	1.76	108.33
GPSSVEDIK	2.42	2	931.47289	-0.28	58.27

Supplementary Table S6. Tumor type abbreviations as shown in Figure 6 and Supplementary Figures S8-S9.

ACC	Adrenocortical carcinoma
BLCA	Bladder Urothelial Carcinoma
BRCA	Breast invasive carcinoma
CESC	Cervical squamous cell carcinoma and endocervical adenocarcinoma
CHOL	Cholangio carcinoma
COAD	Colon adenocarcinoma
DLBC	Lymphoid Neoplasm Diffuse Large B-cell Lymphoma
ESCA	Esophageal carcinoma
GBM	Glioblastoma multiforme
HNSC	Head and Neck squamous cell carcinoma
KICH	Kidney Chromophobe
KIRC	Kidney renal clear cell carcinoma
KIRP	Kidney renal papillary cell carcinoma
LAML	Acute Myeloid Leukemia
LGG	Brain Lower Grade Glioma
LIHC	Liver hepatocellular carcinoma
LUAD	Lung adenocarcinoma
LUSC	Lung squamous cell carcinoma
MESO	Mesothelioma
OV	Ovarian serous cystadenocarcinoma
PAAD	Pancreatic adenocarcinoma
PCPG	Pheochromocytoma and Paraganglioma
PRAD	Prostate adenocarcinoma
READ	Rectum adenocarcinoma
SARC	Sarcoma
SKCM	Skin Cutaneous Melanoma
STAD	Stomach adenocarcinoma
TGCT	Testicular Germ Cell Tumors
THCA	Thyroid carcinoma
THYM	Thymoma
UCEC	Uterine Corpus Endometrial Carcinoma
UCS	Uterine Carcinosarcoma
UVM	Uveal Melanoma

Supplementary Table S7. The 23 genes comprising the hypoxia signature as identified with STRING (KEGG pathway analysis) from an original dataset of 200 genes (GSEA; hallmark hypoxia).

Gene Name	NCBI (Entrez) Gene Id	Gene Description
ALDOA	226	Aldolase, fructose-bisphosphate A
BCL2	596	BCL2 apoptosis regulator
CDKN1A	1026	Cyclin dependent kinase inhibitor 1A
CDKN1B	1027	Cyclin dependent kinase inhibitor 1B
EGFR	1956	Epidermal growth factor receptor
ENO1	2023	Enolase 1
ENO2	2026	Enolase 2
ENO3	2027	Enolase 3
GAPDH	2597	Glyceraldehyde-3-phosphate dehydrogenase
HK1	3098	Hexokinase 1
HK2	3099	Hexokinase 2
HMOX1	3162	Heme oxygenase 1
IL6	3569	Interleukin 6
LDHA	3939	Lactate dehydrogenase A
PDK1	5163	Pyruvate dehydrogenase kinase 1
PFKFB3	5209	6-phosphofructo-2-kinase/fructose-2,6-biphosphatase 3
PFKL	5211	Phosphofructokinase, liver type
PGK1	5230	Phosphoglycerate kinase 1
PRKCA	5578	Protein kinase C alpha
SERPINE1	5054	Serpin family E member 1
SLC2A1	6513	Solute carrier family 2 member 1
VEGFA	7422	Vascular endothelial growth factor A
VHL	7428	Von Hippel-Lindau tumor suppressor

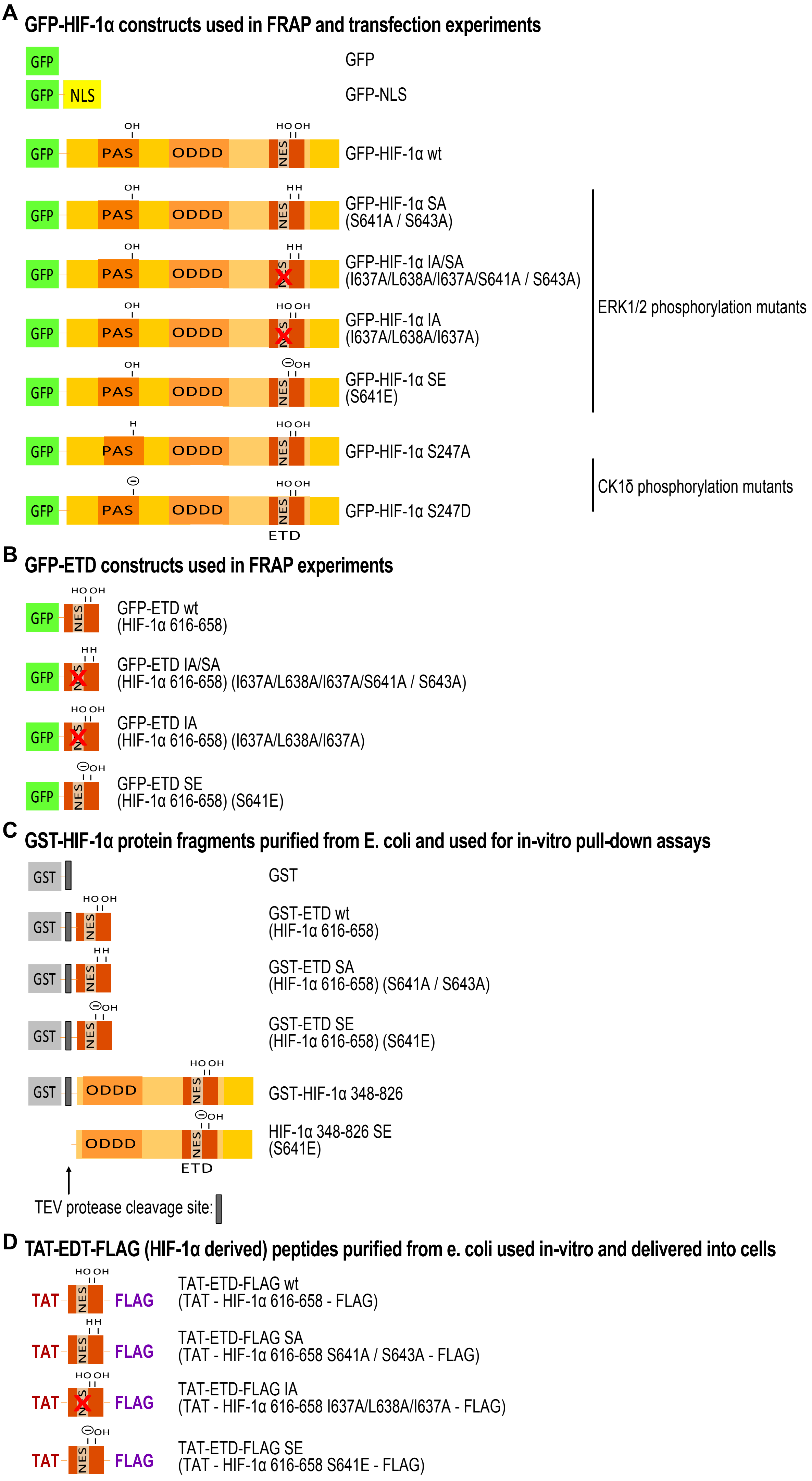

SUP. FIGURE S1

Sup. Figure S1. Schematic representation of HIF-1α forms used in this study. (**A**) Depiction of full-length GFP-tagged HIF-1α forms used in transient transfection experiments (**Fig. 1A**; FRAP, **Fig. 2B,E**). (**B**) De piction of GFP-tagged ETD forms used in transient transfection FRAP experiments (**Fig. 1B**). (**C**) Depiction of GST-tagged ETD or HIF-1α (348-826) forms purified from E. coli (**Sup. Fig. 4A,B**; BAITS) and used for in-vitro pull down assays (**Fig. 1C,E,G**; **Sup. Fig. 4A,B**). (**C**) Depiction of cell permeable TAT-ETD-FLAG peptide forms purified from E. coli (**Sup. Fig. 4C**) and used in-vitro (**Fig. 1H**) or delivered into cells (**Fig. 5F**).

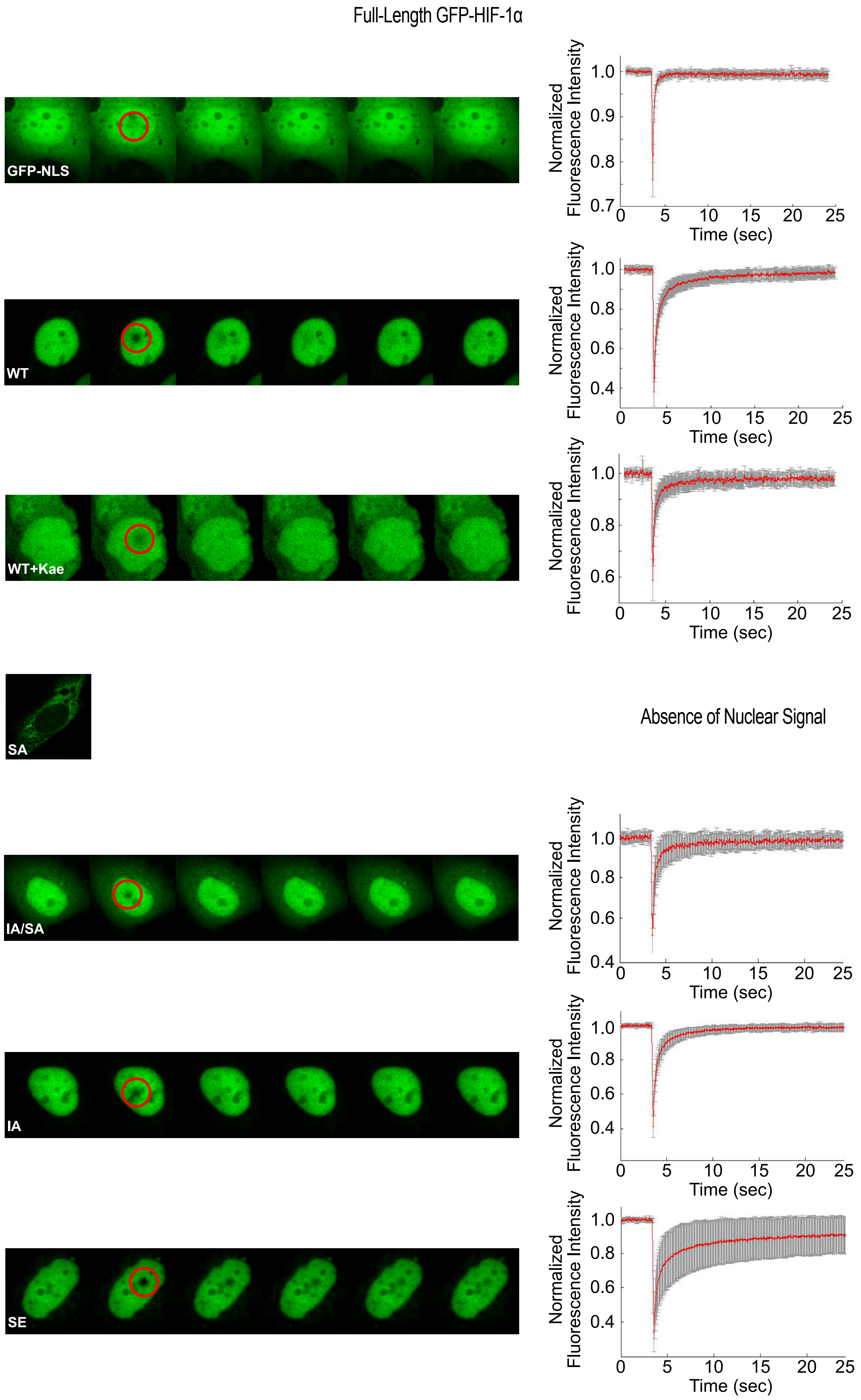

SUP. FIGURE S2

Sup. Figure S2. HIF-1α phosphorylation by ERK1/2 stimulates HIF-1α binding to chromatin components. Left: Representative time-lapse confocal fluorescence microscopy images of live Huh7 cells expressing the indicated full-length GFP-tagged HIF-1α mutant forms. WT+Kae indicate cells treated with kaempferol for 4h. The GFP-HIF-1α SA mutant form was not analyzed as its signal was cytoplasmic. Right: Graphs represent the mean normalized FRAP recovery curves over time for the different GFP-HIF-1α constructs (as indicated). Grey vertical lines represent ± s.d. of mean normalized curves.

GFP-ETDs

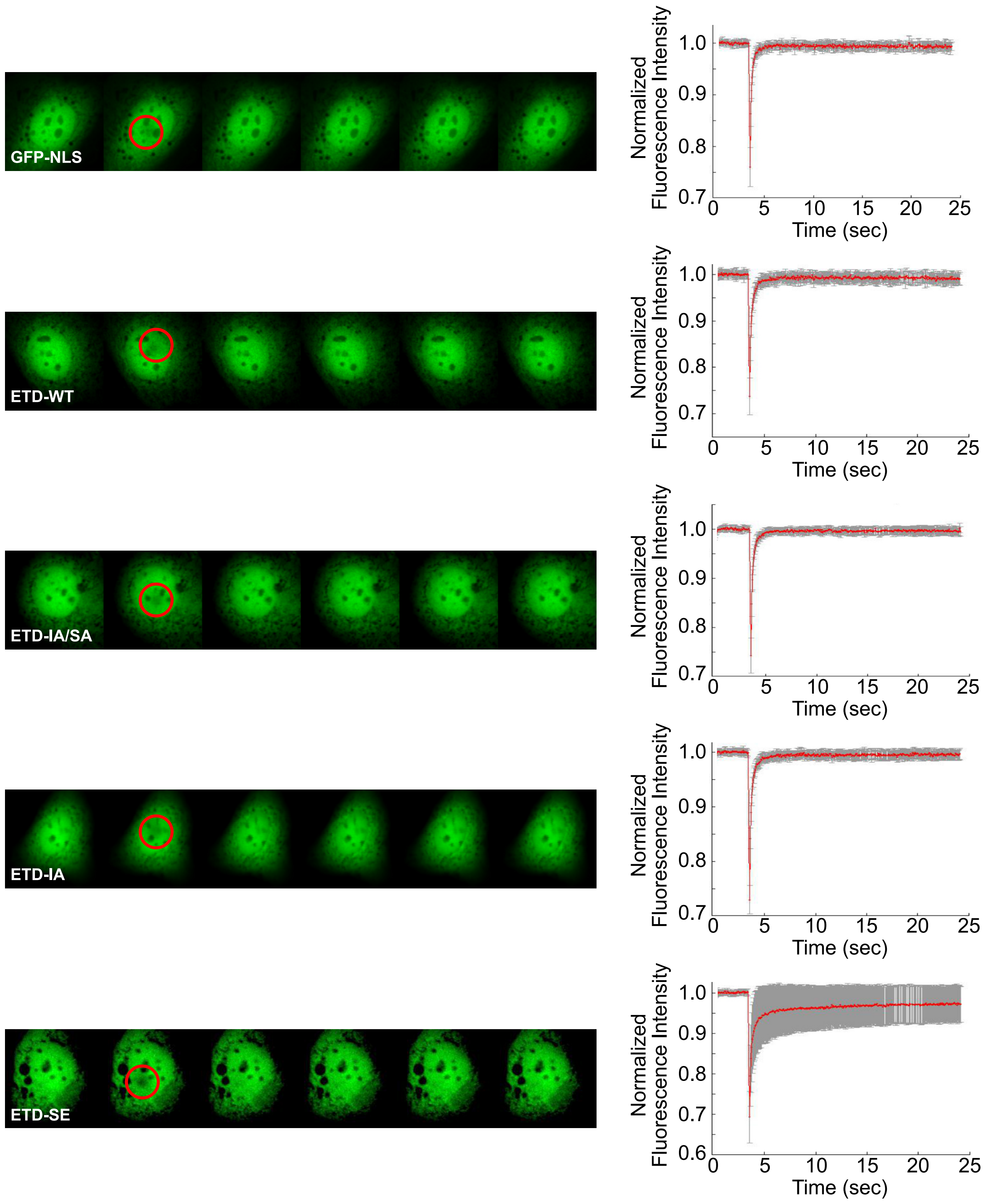

SUP. FIGURE S3

Sup. Figure S3. HIF-1α phosphorylation by ERK1/2 stimulates HIF-1α binding to chromatin components Left: Representative time-lapse confocal fluorescence microscopy images of live Huh7 cells expressing GFP-NLS or the indicated GFP-ETD peptide forms. Right: Graphs represent the mean normalized FRAP recovery curves over time for the different GFP-ETD constructs (as indicated). Grey vertical lines represent ± s.d. of mean normalized curves.

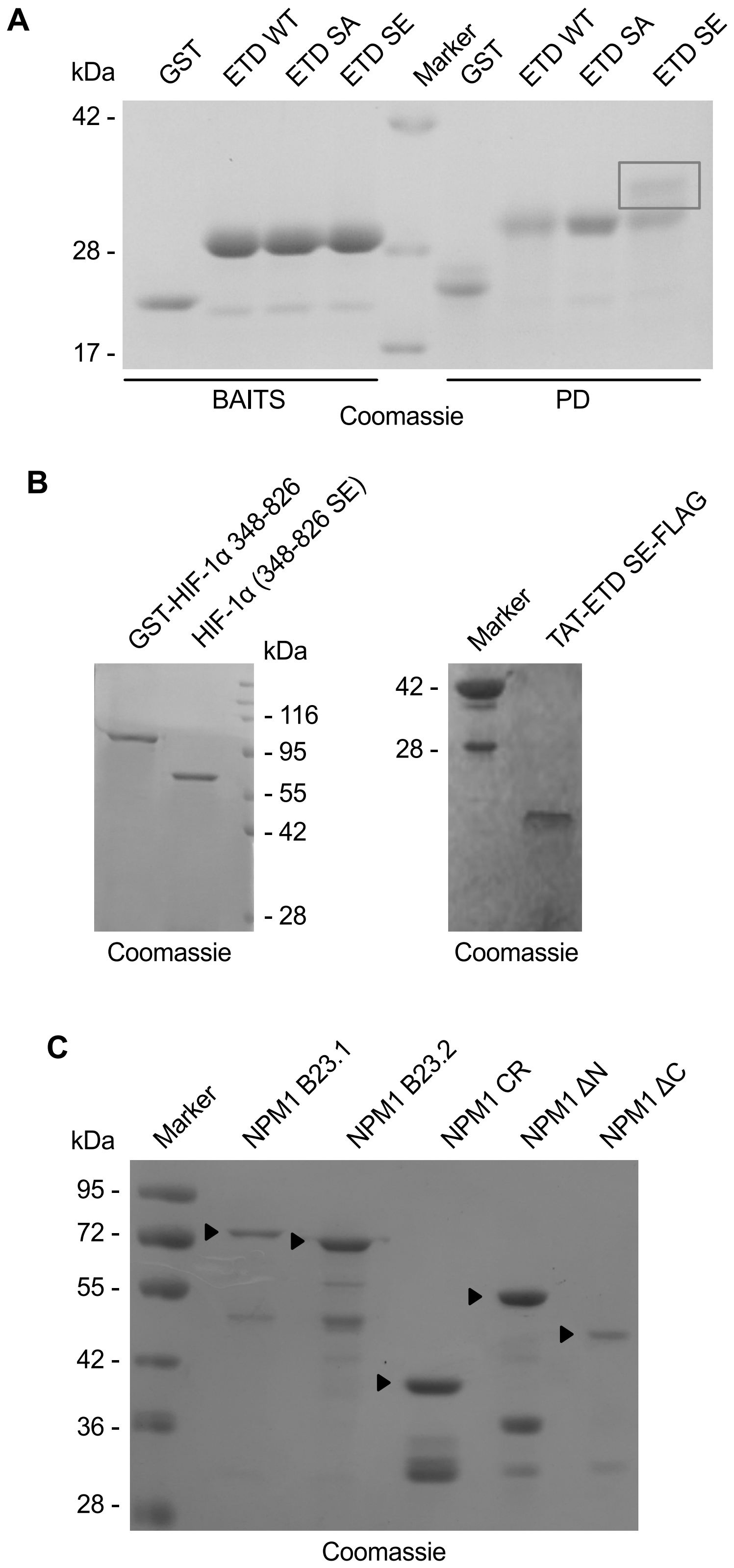
A
kDa
GST
ETD WT
ETD SA
ETD SE
Marker
GST
ETD WT
ETD SA
ETD SE
42 -
28 -
17 -
BAITS
Coomassie
PD
B
GST-HIF-1α 348-826
HIF-1α (348-826 SE)
kDa
- 116
- 95
- 55
- 42
- 28
Coomassie
Marker
TAT-ETD SE-FLAG
42 -
28 -
Coomassie
C
Marker
NPM1 B23.1
NPM1 B23.2
NPM1 CR
NPM1 ΔN
NPM1 ΔC
kDa
95 -
72 -
55 -
42 -
36 -
28 -
Coomassie

SUP. FIGURE 4

Sup. Figure S4. SDS-PAGE analysis of recombinant HIF-1α and NPM1 forms used in this study. (**A**) Recombinant GST and GST-ETD WT, SA, SE purified from E. coli (BAITS) and used in in-vitro pull down assays from HeLa cells (PD); Box corresponds to the ~36kD protein band co-precipitating with GST-ETD SE (also **Fig. 1C**). (**B**) Left: Recombinant GST-HIF-1α (348-826) and HIF-1α (348-826) SE purified from E. coli and used in pull down assays shown in **Fig. 1E,G** respectively. Right: Recombinant TAT-ETD SE-FLAG (HIF-1α 616-658) purified from E. coli and used in pull down assays shown in **Fig. 1H**. (**C**) Recombinant GST-NPM1 forms (as indicated) purified from E. coli and used in pull down assays shown in **Fig. 1E,G,H**.

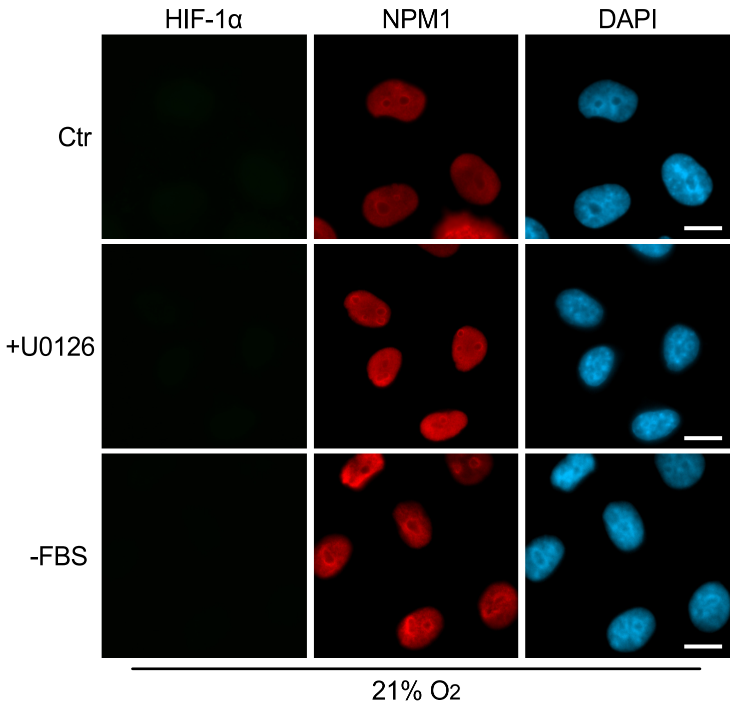

SUP. FIGURE S5

Sup. Figure S5. NPM1 immunofluorescence under normoxic conditions. Immunofluorescence microscopy images of HeLa S3 cells grown at 21% O_2 for 16 h with no further treatment (Ctr) or treated with 5 µM U0126 (+U0126) or deprived of serum (-FBS), using antibodies against HIF-1α (Green) and NPM1 (Red). DAPI staining was used to show nuclei (Cyan). Scale bars: 10 µM. Notice that NPM1 displays similar localization under all conditions.

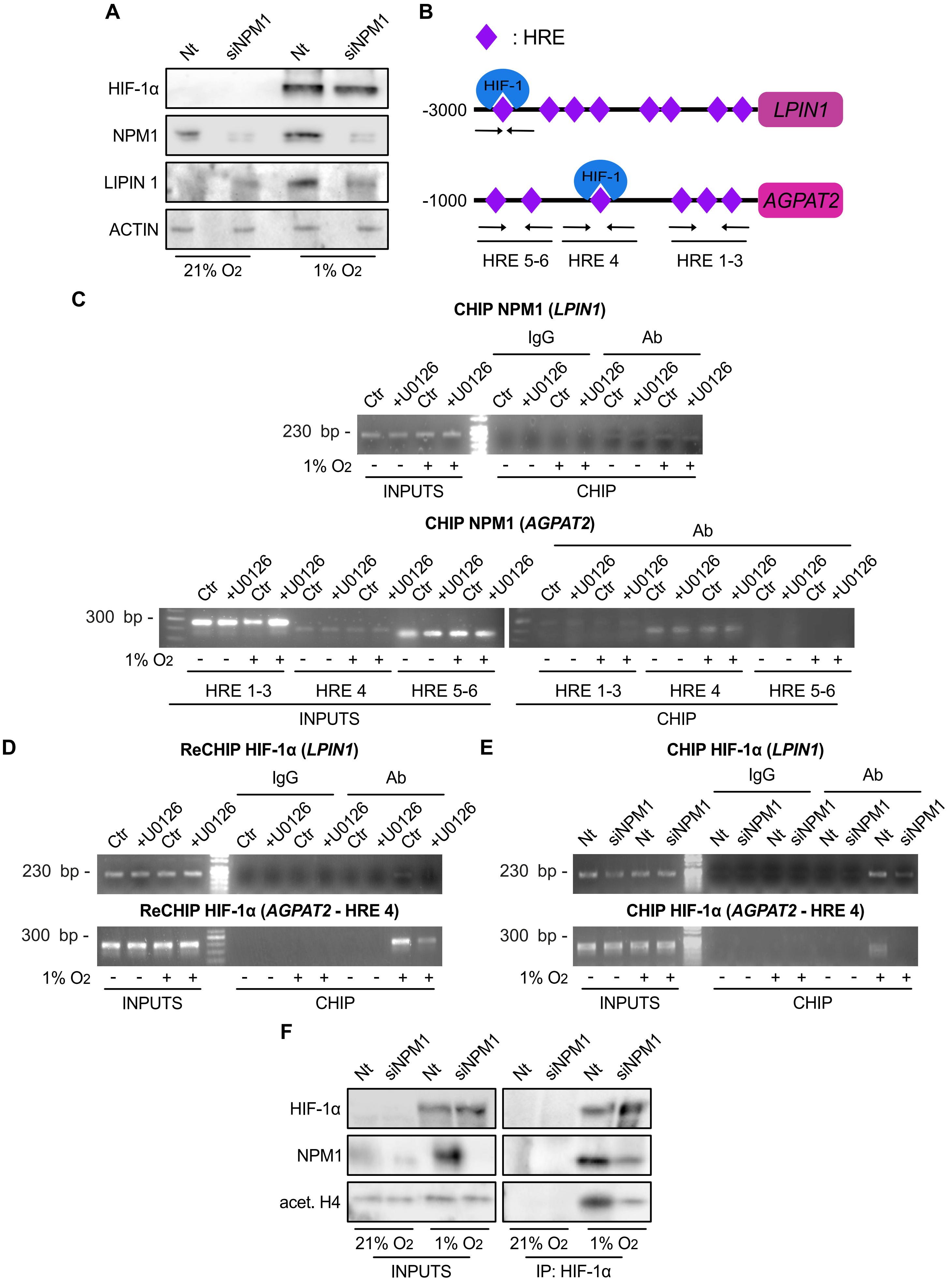

SUP. FIGURE S6

Sup. Figure S6. NPM1 is essential for HIF-1 transcriptional activity and stable binding to HRE and components of open chromatin. (**A**) Immunoblotting analysis of soluble protein extracts from HeLa cells treated as in (**Fig. 3C**) using antibodies against the indicated proteins. (**B**) Schematic representation of *LPIN1* and *AGPAT2* promoter regions indicating the potential HRE sequences (Diamonds). Arrows indicate the set of primers for DNA amplification after ChIP (Suppl. Table S7). The presence of HIF-1 (Blue Circle) indicates the functional HRE sequences as previously identified (Mylonis et al. 2012; Triantafyllou et al. 2018). (**C-E**) Analysis by agarose gel electrophoresis of amplified PCR products from: (**C**) Sequential chromatin immunoprecipitation using initially anti-NPM1 antibodies (ChIP) and followed by (**D**) second immunoprecipitation with anti-HIF-1α antibodies (ReChIP) in extracts of Huh7 cells grown at 21% or 1% O_2 for 24h with or without 10 μM U0126. (**E**) Chromatin immunoprecipitation with anti-HIF-1α antibodies from Huh7 cells treated with control (Nt) or NPM1 siRNA (siNPM1) for 24h and incubated at 21% or 1% O_2 for 8h. (**C-E**) The precipitated DNA was amplified by PCR using primers specific for the distal HRE of *LPIN1* promoter (upper panels) or areas containing the different HREs of the *AGPAT2* promoter (lower panels; as indicated). (**F**) Immunoblotting analysis of soluble extracts (INPUTS) and anti-HIF-1α immunoprecipitates (IP) from HeLa cells treated with control (Nt) or NPM1 siRNA (siNPM1) for 24h and then incubated at 21% or 1% O_2 for 16h.

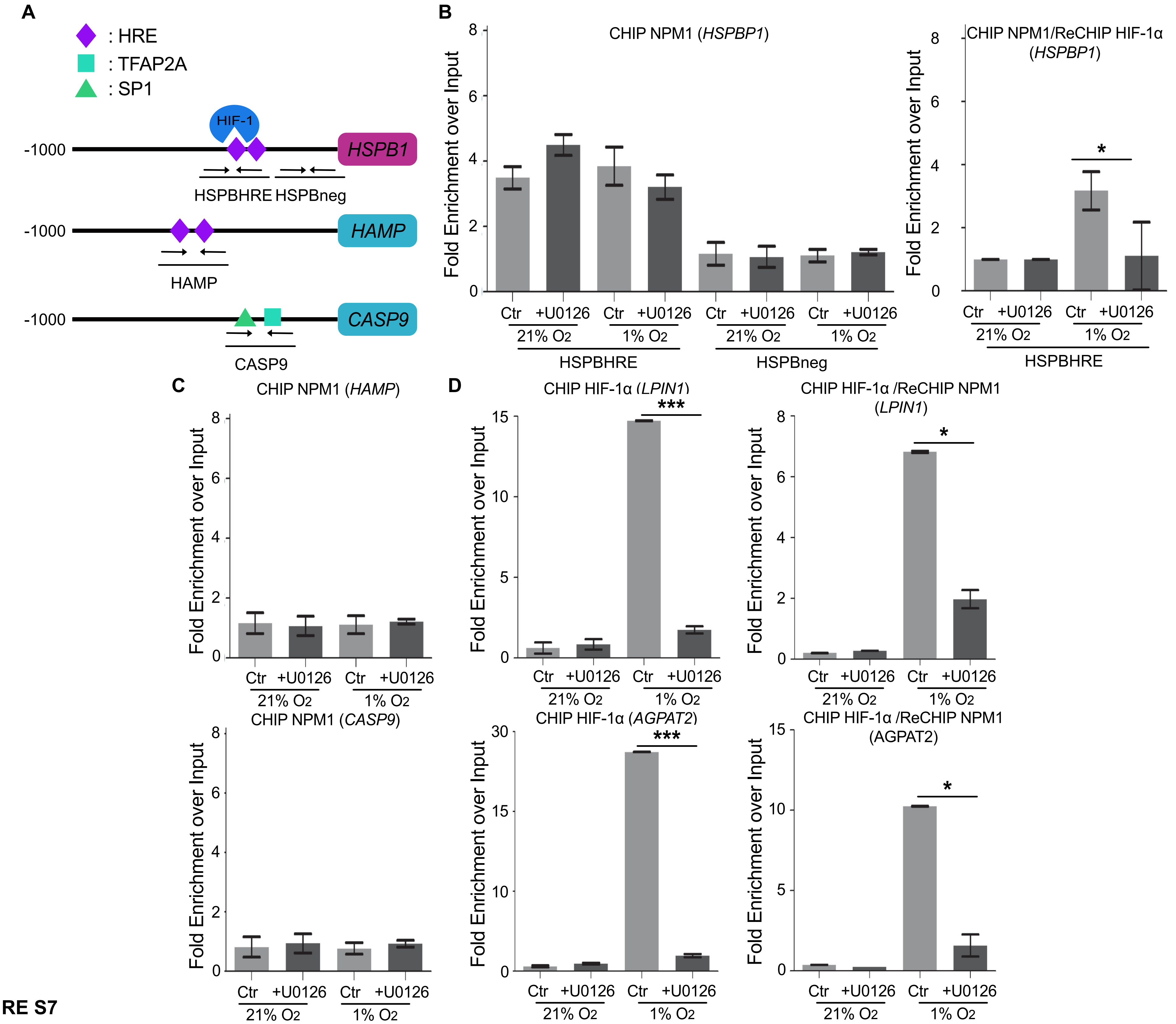

SUP. FIGURE S7

Sup. Figure S7. NPM1 occupies functional HRE sequences. (**A**) Schematic representation of *HSPB1*, *HAMP*, and *CASP9* promoter regions indicating the potential HRE (Diamonds), TFAP2A (square) or SP1 (Triangle) sequences. Arrows indicate the set of primers for DNA amplification after ChIP (Suppl. Table S7). The presence of HIF-1 (Blue Circle) indicates the functional HRE sequences as previously reported (Whitlock et al. 2005). (**B**) RT-PCR analysis of sequential chromatin immunoprecipitation using initially anti-NPM1 antibodies (ChIP; left panel) and followed by second immunoprecipitation with anti-HIF-1α antibodies (ReChIP; right panel) in extracts of Huh7 cells grown at 21% or 1% O_2 for 24h with or without 10 μM U0126. Left panel shows analysis using primers for the areas of the *HSPB1* promoter containing known HIF-1 binding sites (HSPBHRE) or neighboring non-HRE sequences (HSPBneg; shown in A) (**C**) RT-PCR analysis of chromatin immunoprecipitation using anti-NPM1 antibodies (ChIP) in extracts of Huh7 cells grown at 21% or 1% O_2 for 24h with or without 10 μM U0126. Upper panel shows analysis using primers for the areas of the *HAMP* promoter containing HRE-like sequences (HAMP; shown in A) (Braliou et al. 2008). Lower panel shows analysis using primers for the areas of the *CASP9* promoter containing TFAP2A and SP1 sequences (CASP9; shown in A) (Orso et al. 2010). (**D**) RT-PCR analysis of sequential chromatin immunoprecipitation using initially anti-HIF-1α antibodies (ChIP; left panel) and followed by second immunoprecipitation with anti-NPM1 antibodies (ReChIP; right panel) in extracts of Huh7 cells grown at 21% or 1% O_2 for 24h with or without 10 μM U0126. Upper panels show analysis using primers for the area of the *LPIN1* promoter containing a known HIF-1 binding site (**Supl. Fig. S6B**). Bottom panels show analysis using primers for the area of the *AGPAT2* promoter containing a known HIF-1 binding site (HRE4) (**Supl. Fig. S6B**)

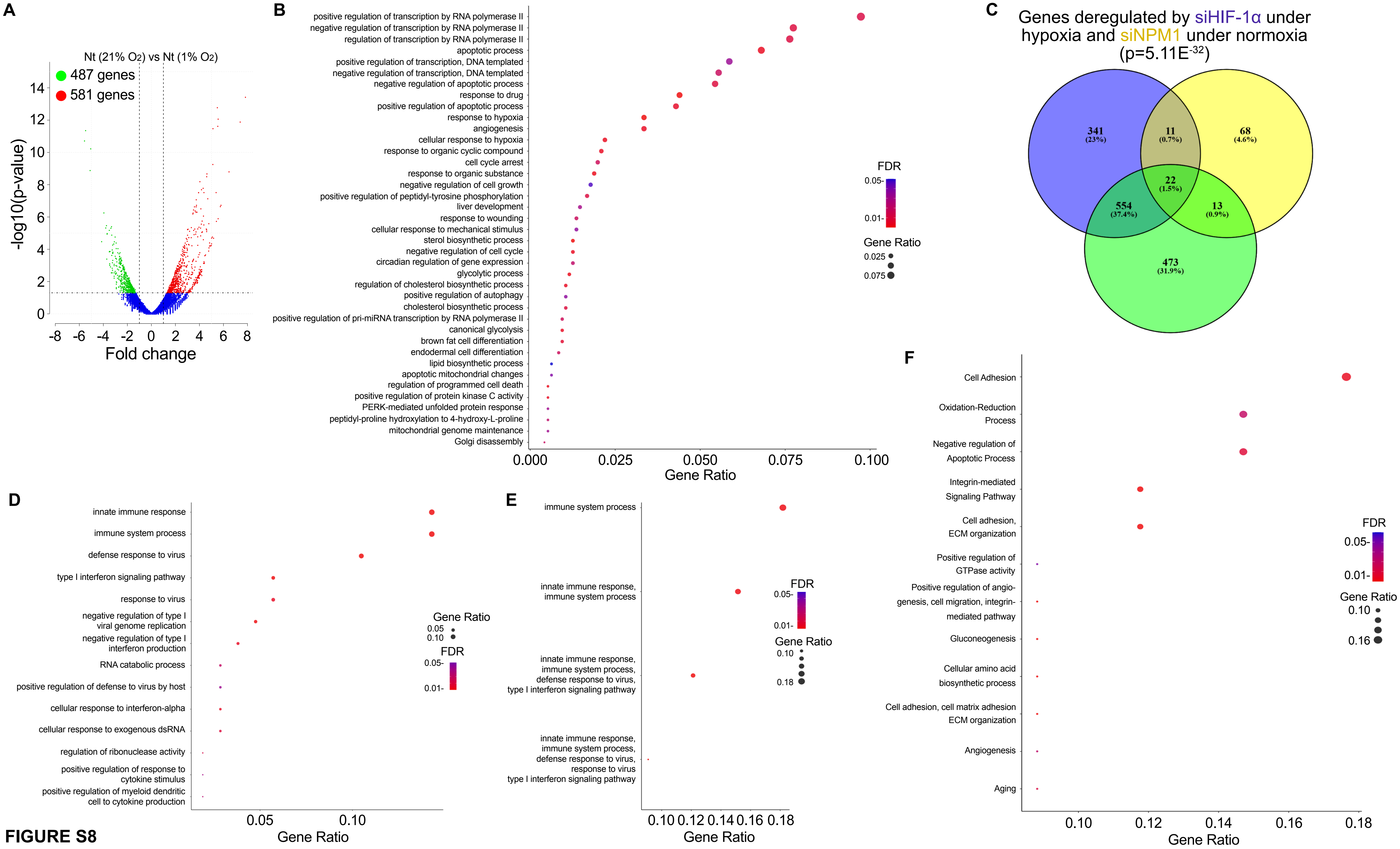

SUP. FIGURE S8

Sup. Figure S8. (**A**) Volcano plot of genes showing significantly different expression levels in cells incubated under normoxia (21% O_2) or hypoxia (1% O_2). Normalized enrichment score from Gene Set Enrichment Analysis is shown. P-value < 0.05. (**B**) Dot plot of GO BP ontology analysis of genes deregulated by hypoxic treatment. (**C**) Venn diagram representing the number of genes significantly deregulated after HIF-1α silencing under hypoxia (Magenta) or after NPM1 silencing under normoxia (Yellow) or after hypoxic treatment alone (Green). (**D**) Dotplot of GO BP ontology analysis of genes with deregulated expression in cells treated with NPM1 siRNA under normoxia. (**E**) Dotplot of GO BP ontology analysis of genes commonly deregulated in cells treated with HIF-1α siRNA under hypoxia and NPM1 siRNA under normoxia (33 genes as represented in (**C**)). (**F**) Dot plot of GO BP ontology analysis of common genes deregulated by either HIF-1α or NPM1 silencing as represented in Figure **4B**.

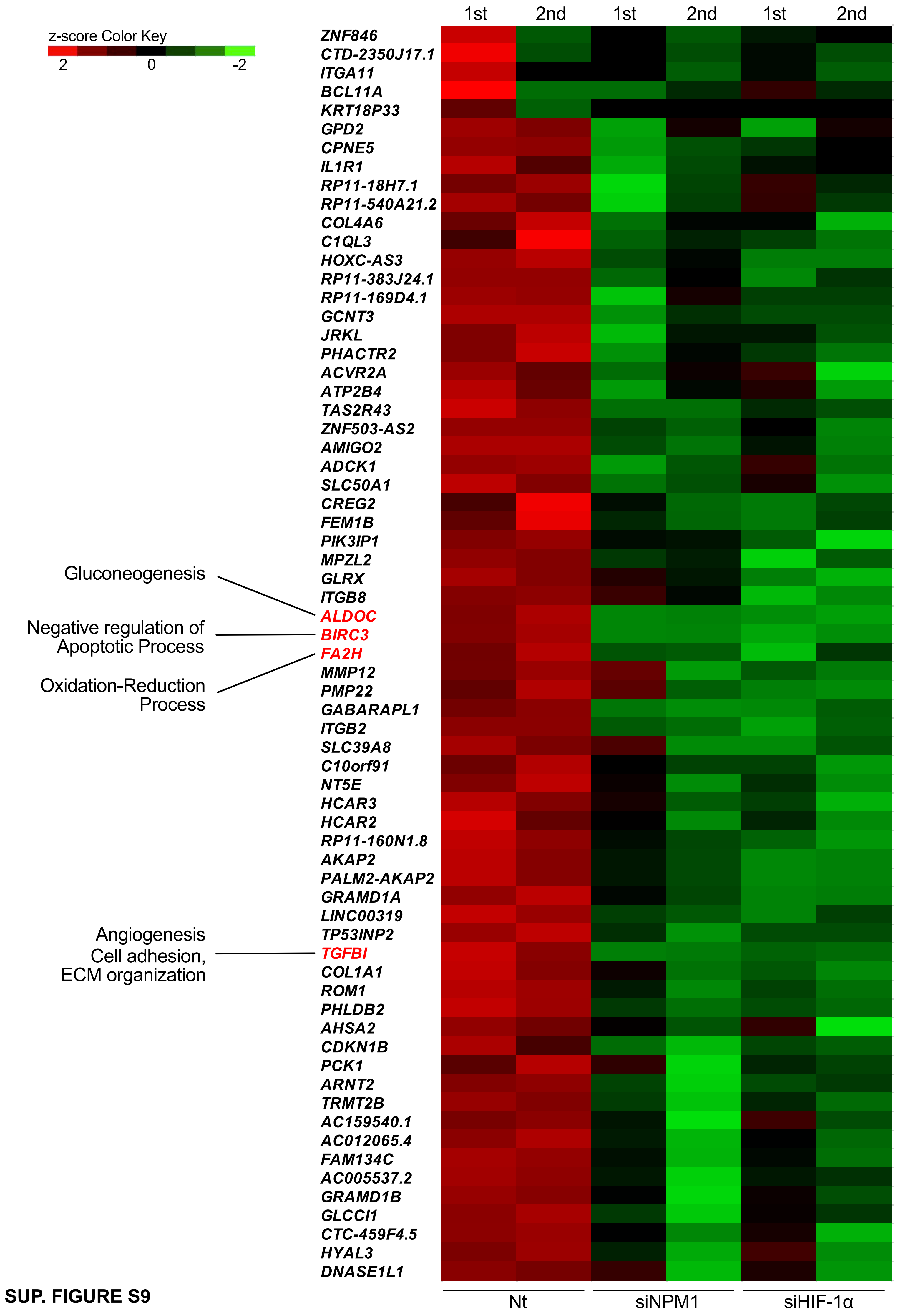

SUP. FIGURE S9

Sup. Figure S9. The 67 genes co-upregulated by NPM1 and HIF-1α under hypoxia. Heatmap of normalized counts for genes (as indicated) in control (Nt) or siRNA-treated HeLa S3 cells (as indicated) and processed as in Figure **4**. Counts from both biological replicates are represented (1st or 2nd; as indicated). Names of genes with expression levels verified by RT-PCR are depicted in red colour (including gene ontology).

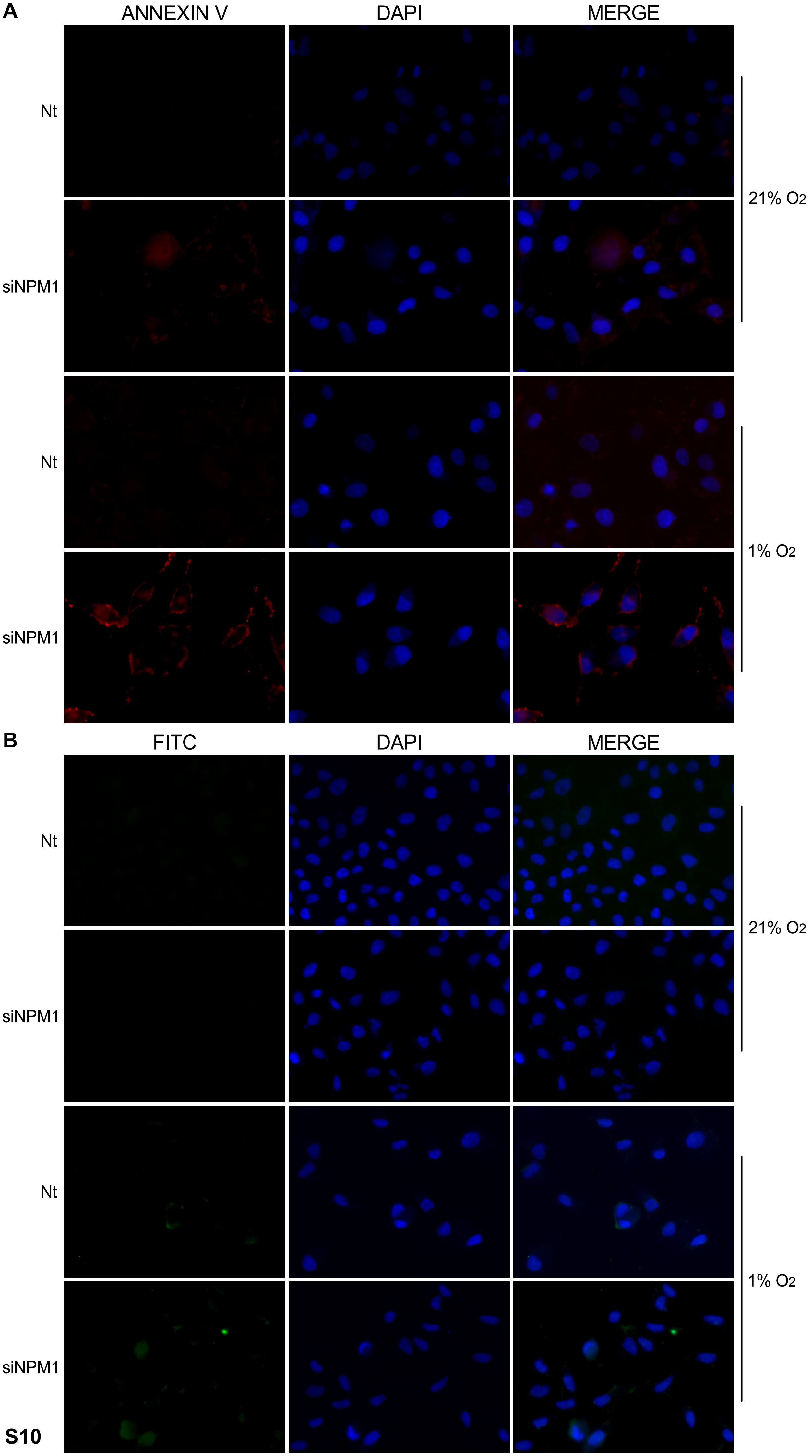

SUP. FIGURE S10

Sup. Figure S10. Annexin V and TUNEL staining in HeLa cells. Representative fluorescence microscopy images of HeLa S3 cells transfected with control (Nt) or NPM1 siRNA (siNPM1) and grown at 21% or 1% O_2 for 24h. Positive Annexin V staining is shown by red fluorescent signal in (A) and green (FITC) fluorescent signal represents TUNEL positive cells in (B). DAPI staining was used to show nuclei (Blue).

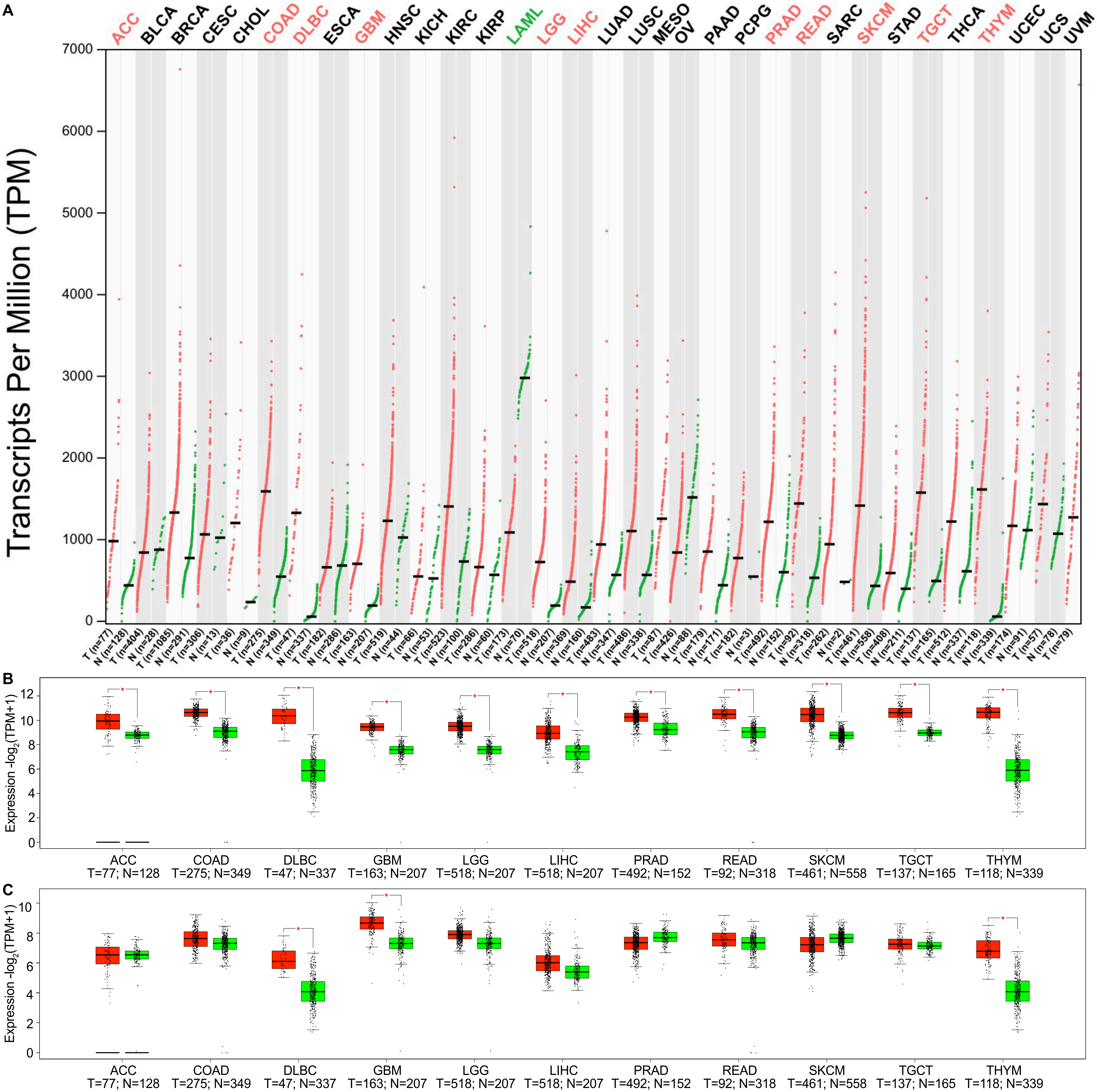

SUP. FIGURE S11

Sup. Figure S11. (**A**) Dot plot of *NPM1* expression profile across all publicly available samples of tumor types (red dots) and their paired normal tissues (green dots) as analyzed on the GEPIA2 platform using the TCGA database. Each dot represents *NPM1* expression in a single patient sample. Colored abbreviations of cancer type names depict significantly higher NPM*1* expression in tumor (red) or normal samples (green); n: number of samples in each tumor type. (**B**) Boxplots showing significantly higher *NPM1* expression (P<0.05) in 11 different types of human cancer (Red; T) compared to paired healthy tissue samples (Green; N). Each dot represents a different patient sample. (**C**) Boxplots comparing the expression of the 67 genes commonly upregulated by HIF-1 and NPM1 under hypoxia in HeLa cells (this study; Supplementary Fig. S6) between the 11 different types of human cancer (Red; T) shown in (**B**) and their corresponding paired normal tissue (Green; N) samples. Each dot represents a sample (*P<0.05).

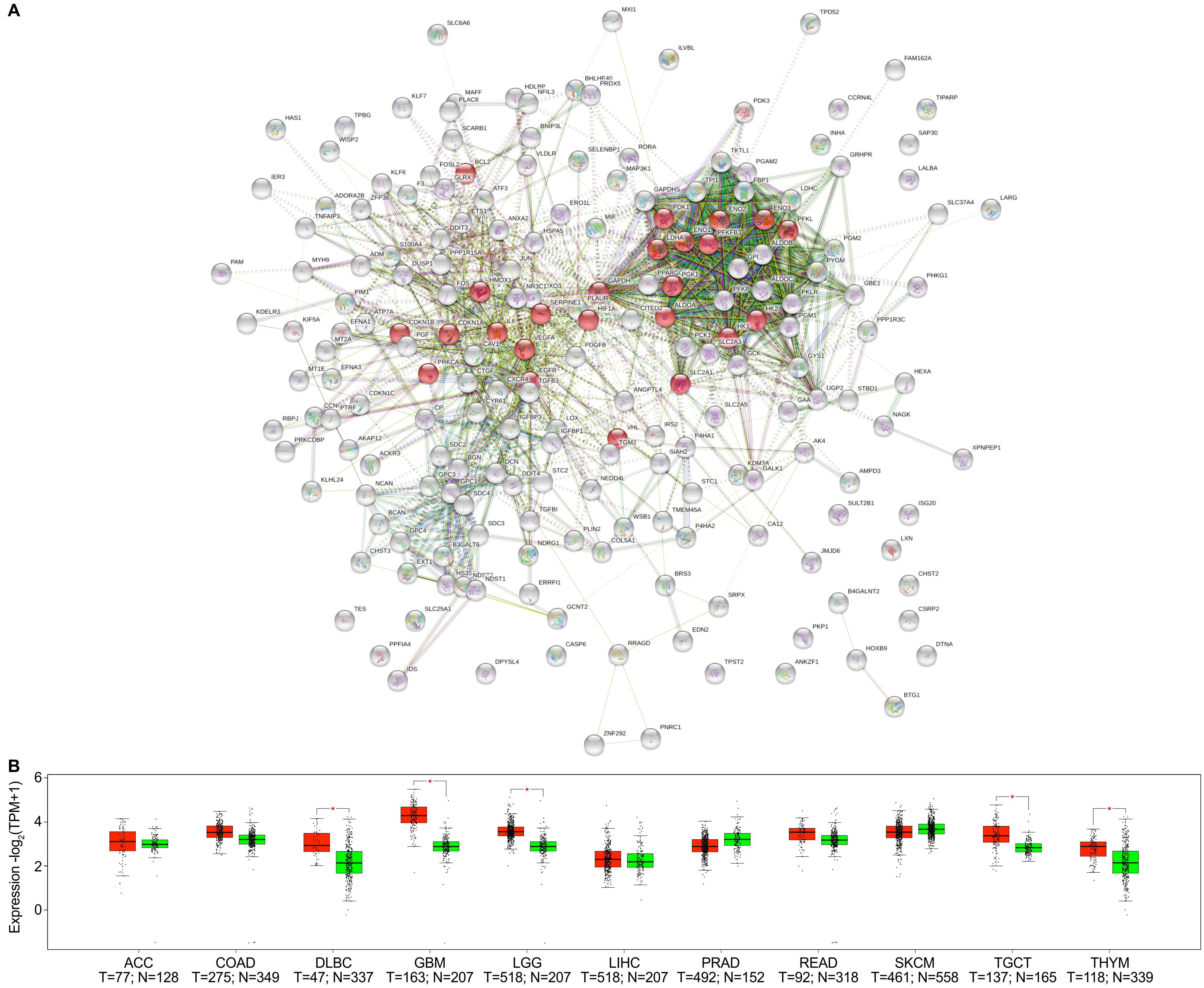

SUP. FIGURE S12

Sup. Figure S12. (**A**) STRING analysis showing genes with strong association with the HIF-1 signaling pathway (KEGG pathway analysis). 23 genes in addition to HIF-1 were identified and are highlighted in red. (**B**) Boxplots comparing the expression of a hypoxia 23-gene signature (**A and supplementary Table S6**) between the 11 different types of human cancer (Red; T) shown in (**supplementary Fig. S11B**) and their corresponding paired normal tissue (Green; N) samples. Each dot represents a sample (*P<0.05).
